# Regulation of the Flavonoid Biosynthesis Pathway Genes in Purple and Black Grains of *Hordeum vulgare*

**DOI:** 10.1371/journal.pone.0163782

**Published:** 2016-10-05

**Authors:** Olesya Yu. Shoeva, Hans-Peter Mock, Tatjana V. Kukoeva, Andreas Börner, Elena K. Khlestkina

**Affiliations:** 1 Institute of Cytology and Genetics (ICG), Siberian Branch, Russian Academy of Sciences, Novosibirsk, Russia; 2 Leibniz Institute of Plant Genetics and Crop Plant Research (IPK), Gatersleben, Germany; 3 Food Security Research Center, Novosibirsk State University (NSU), Novosibirsk, Russia; Institute for Resistance Research and Stress Tolerance, GERMANY

## Abstract

Barley grain at maturity can have yellow, purple, blue, and black pigmentations which are suggested to play a protective role under stress conditions. The first three types of the colors are caused by phenolic compounds flavonoids; the last one is caused by phytomelanins, oxidized and polymerized phenolic compounds. Although the genetic basis of the flavonoid biosynthesis pathway in barley has been thoroughly studied, there is no data yet on its regulation in purple and black barley grains. In the current study, genetic model of *Hordeum vulgare* ‘Bowman’ near-isogenic lines (NILs) was used to investigate the regulation of the flavonoid biosynthesis in white, purple, and black barley grains. Microsatellite genotyping revealed donor segments in the purple- and black-grained lines on chromosomes 2H (in region of the *Ant2* gene determining purple color of grains) and 1H (in region of the *Blp* gene determining black lemma and pericarp), respectively. The isolated dominant *Ant2* allele of the purple-grained line has high level of sequence similarity with the recessive Bowman’s *ant2* in coding region, whereas an insertion of 179 bp was detected in promoter region of *ant2*. This structural divergence between *Ant2* and *ant2* alleles may underlie their different expression in grain pericarp: Bowman’s *Ant2* is not transcribed, whereas it was up-regulated in the purple-grained line with coordinately co-expressed flavonoid biosynthesis structural genes (*Chs*, *Chi*, *F3h*, *F3’h*, *Dfr*, *Ans*). This led to total anthocyain content increase in purple-grained line identified by ultra-performance liquid chromatography (HPLC). Collectively, these results proved the regulatory function of the *Ant2* gene in anthocyanin biosynthesis in barley grain pericarp. In the black-grained line, the specific transcriptional regulation of the flavonoid biosynthesis pathway genes was not detected, suggesting that flavonoid pigments are not involved in development of black lemma and pericarp trait.

## Introduction

**Fig 1:**
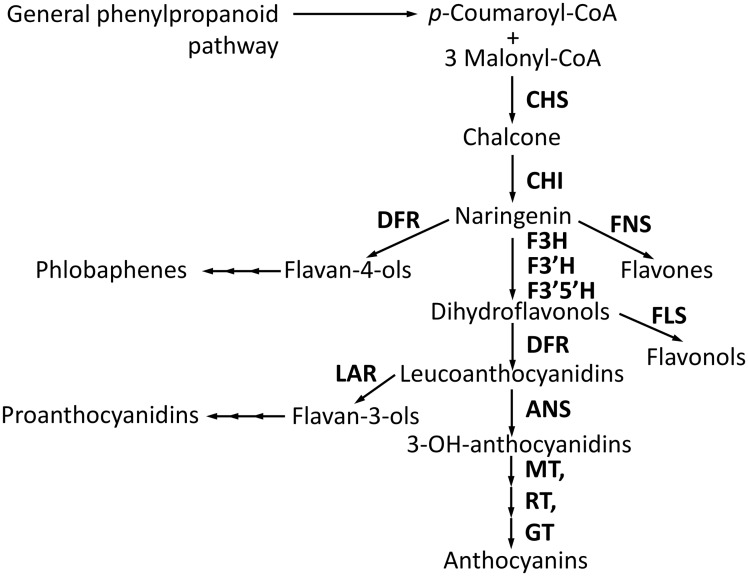
Flavonoid biosynthetic pathway in plants. The enzymes are: CHS (chalcone synthase); CHI (chalcone-flavanone isomerase); F3H (flavanone 3-hydroxylase); FLS (flavonol synthase); FNS (flavone synthase); F3’H (flavonoid 3’-hydroxylase); F3’5’H (flavonoid 3’,5’-hydroxylase); DFR (dihydroflavonol 4-reductase); ANS (anthocyanidin synthase); GT (glycosyltransferase); MT (methyltransferase), RT (rhamnosyltransferase); and LAR (leucoanthocyanidin reductase).

Barley (*Hordeum vulgare* L., *2n* = *2x* = 14, HH) grain at maturity may have different pigmentations. The most studied ones are yellow, purple, red, and blue caused by different subgroups of flavonoid compounds (Fig 1). The yellow color is due to proanthocyanidins synthesized in seed coat (testa layer) [1]; purple and red pigments are anthocyanins synthesized in pericarp and glumes [2]; blue color is caused by anthocyanins synthesized in aleurone layer of the grain [2]. In white barley grains, the pigments are absent. Scientific interest to the pigments is caused by their protective functions under different environments [3] as well as their undoubted health benefit [4]. In barley, flavonoid biosynthesis metabolic pathway is well studied [5]: structural genes encoding enzymes of the pathway, as well as regulatory genes, predetermining temporal and spatial patterns of the structural gene expressions have been described (S1 Table).

Chalcone synthase (CHS) represents in the barley genome a gene family with at least seven copies [6]. One copy of the gene has been isolated by heterologous hybridization method using a cDNA clone from *Antirrhinum majus* as a probe [7, 8]. Another copy of the gene with unusual substrate preference has been identified in pathogen-induced barley leaves cDNA library [6]. One of the *Chs* gene copies has been mapped to the short arm of chromosome 1H using gDNA clone from barley genome as a probe [9]. Three non-overlapping genetic markers for the *Chs* gene have been mapped to chromosomes 1HS, 1HL, and 6HS [10]. The chalcone-flavanone isomerase gene (*Chi*) has been identified and mapped to the long arm of chromosome 5H using Southern blot technic with nucleotide sequence of the maize *Chi* gene as a probe [11]. Flavanone 3-hydroxylase gene (*F3h*) has been identified in cDNA barley library using cDNA probe from *A. majus* [12]. The gene has been mapped to chromosome 2HL [10, 13]. A full-length cDNA copy of the barley dihydroflavanol reductase gene (*Dfr*) has been isolated from kernel-specific cDNA library using cDNA of the maize *Dfr* gene as a probe [14]. The gene has been localized on the long arm of chromosome 3H [10]. Possible nucleotide sequence for the barley leucoanthocyanidin reductase (LAR) has been identified among plant expressed sequence tag (EST) sequences by querying the EST database with the Desmodium protein sequence using the tBLASTn procedure [15]. The *Lar* gene has not been located in barley genome yet. The full-length nucleotide sequence of the gene for the UDP glucose:flavonol 3-*O*-glucosyltransferase (UFGT) has been isolated from barley genome by Southern blot method with the nucleotide sequence of the maize *Ufgt* gene as a probe. The gene has been mapped to the short arm of chromosome 7H [16]. The full-length nucleotide sequences of the flavonoid 3’-hydroxylase (*F3’h*) and anthocyanidin synthase (*Ans*) genes were identified in the current study (S1 and S2 Files, respectively). The identified contigs of the *F3’h* and *Ans* genes are localized on chromosomes 1H and 5HL, respectively.

Among barley flavonoid biosynthesis regulatory genes full-length nucleotide sequences have been recently isolated for the *Ant1* [17, 18], *Ant2* [19] and *Ant28* [20] genes.

In *ant1* mutants, anthocyanin pigments are not observed in stem, auricles, awns, or lemma [21]. The *Ant1* gene encodes for a R2R3 MYB-type transcription factor that activates transcription of the anthocyanin biosynthesis structural genes (*Chi*, *F3h*, *Dfr*, *Ans*) in leaf sheaths [18]. The gene has been located on chromosome 7HS [17, 22].

The *Pre2* gene controls purple lemma and pericarp trait development in barley [23]. The gene has been mapped to the short arm of chromosome 2H [24], very close to the microsatellite marker linked with the *Ant2* gene, which encodes for a transcription factor with a basic Helix-Loop-Helix domain (bHLH) and is suggested to be a regulatory gene for the anthocyanin biosynthesis pathway [19, 25]. In *ant2* mutants, anthocyanin pigments are not observed in stem, auricles, awns, or lemma [21]. The *Ant2* gene is expressed in auricles, awns, and lemma [19]; however its influence on the structural genes expression has not been shown yet.

In *ant28* mutants, proanthocyanidins are not synthesized in grain, whereas wild-type level of anthocyanin content is retained in the vegetative tissue [5]. This data in additional to the observation of reduced enzymatic activities of DFR and LAR (which are essential for proanthocyanidin synthesis, Fig 1) in the *ant28* mutant are suggested that *Ant28* is a specific regulator of proanthocyanidin synthesis [5]. The gene has been mapped to the long arm of chromosome 3H [20, 26].

Blue aleurone color occurs in the presence of the five complementary dominant genes *Blx1*, *Blx2*, *Blx3*, *Blx4* and *Blx5* [27]. *Blx1*, *Blx3* and *Blx4* are closely linked to each other and mapped to chromosome 4HL. *Blx2* and *Blx5* have been assigned to chromosome 7HL [27]. The *Blx* genes have not been sequenced yet and their certain functions in the synthesis of blue anthocyanin substances have not been studied thus far.

Barley grain may have also black pigmentation which is caused by phytomelanins synthesized in glumes and/or pericarp [2]. The trait is controlled by the *Blp* locus, mapped to chromosome 1HL [23].

In the current study, we used Bowman’s NILs [28] to reveal the specific features of the anthocyanin biosynthesis regulation in barley grain and to understand the involvement of the flavonoid biosynthesis pathway in development of black lemma and pericarp trait.

## Materials and Methods

### Plant material, DNA/RNA extraction, cDNA synthesis

A set of the barley cultivar Bowman near-isogenic lines (NILs) were used in the study: PLP (NGB22213) having purple pericarp, BLP (NGB20470) having black lemma and pericarp, and cultivar Bowman (NGB22812) having green lemma and uncolored pericarp (S1 Fig). The set of the lines was provided by the Nordic Gene Bank (NGB, www.nordgen.org). The plants were grown using resources of ICG Greenhouse Core Facilities (Novosibirsk, Russia) under 12 h of light per day at 20-25°C. DNA was extracted from fresh leaves of plants following [29]. Lemma and pericarp for RNA extraction were peeled by scalpel from grains at early dough stage of maturity simultaneously for all genotypes. RNA was extracted applying a Zymo Research Plant RNA MiniPrepTM kit (www.zymoresearch.com) followed by DNAse treatment. A 180 ng aliquot of total RNA was converted to single-stranded cDNA via reverse transcription using a Fermentas RevertAid^™^ first strand cDNA synthesis kit (www.thermoscientificbio.com/fermentas/) primed with (dT)_15_ in a 20 *μ*l reaction volume.

### Microsatellite genotyping

Primers to barley microsatellite loci mapped to chromosome 1H (*Xbmac0032*, *Xbmac0063*, *Xbmac0090*, *Xbmac0154*, *Xbmac0211*, *Xbmac0399*, *Xbmag0579*, *Xbmag0872*, *Xgbms0012*, *Xgbms0037*, *Xgbms0054*, *Xgbms0062*, *Xgbms0184* [30, 31]) and 2H (*Hvm23*, *Hvm54*, *Xbmag0125*, *Xbmag0140*, *Xebmac0607* [31]) were kindly provided by Dr Marion Röder (IPK-Gatersleben, Germany). The PCR-conditions were as described in [32]. Amplicons were separated through 5% ACTGene agarose gels (ACTGene, Inc., Piscataway, NJ, USA). Data on marker localization were taken from Barley consensus map, 2007 (chromosome 1H) and Barley consensus map, 2003 (chromosome 2H) deposited at web-site: wheat.pw.usda.gov/GG2/.

### The *Ant2* gene re-sequencing and sequence analysis

The nucleotide sequence of contig 857662 corresponding to the *Ant2* gene of barley cultivar Bowman (S3 File) was found in BARLEX database (http://apex.ipk-gatersleben.de/apex/f?p=284:10 [33]) using nucleotide sequence of the barley *Ant2* gene encoding protein with bHLH domain (GenBank accession HM370298 [19]) as a query. The full-length *Ant2* sequence of the PLP line was re-constructed from a series of the overlapping amplicons covering the relevant stretch of genomic DNA. The amplicons were generated by primer pairs described by Cockram et al. [19] and by overlapping primer pairs covering 5’ regulatory region designed in the current study (S2 Table). The primers were designed using OLISO software [34]. Amplification of gDNA templates was performed in 20 *μ*l PCRs each containing 1 U *Taq* DNA polymerase (Medigen, Novosibirsk, Russia), 1× PCR buffer (Medigen), 1.8 mM MgCl_2_, 0.2 mM dNTP and 0.25 *μ*M of each primer. Amplification program was initiated with a denaturing step of 95°C / 2 min, 45 cycles of 95°C / 45 s, 55°C / 1 min, 72°C / 2 min, and a final extension of 72°C / 10 min. The amplified fragments were purified from a 2% agarose gel, using a DNA Clean kit (Cytokine, St. Petersburg, Russia), and then sequenced in both directions. DNA sequencing was performed by SB RAS Genomics Core Facilities (Novosibirsk, Russia, http://sequest.niboch.nsc.ru). Multiple sequence alignments were carried out using Multalin v5.4.1 software [35]. Secondary structures of the transcription factors were predicted by the Chou & Fasman Secondary Structure Prediction Server (CFSSP, http://cho-fas.sourceforge.net/index.php [36]). Structural elements of promoters were identified using the PLACE database (http://www.dna.affrc.go.jp/PLACE/signalscan.html [37, 38]).

### qRT-PCR

qRT-PCR was performed with the primers amplifying parts of the flavonoid biosynthesis structural genes *Chs*, *Chi*, *F3h*, *Dfr* [18], *F3’h*, *Ans*, and regulatory gene *Ant2* (F-SSR/R-SSR [19]). *F3’h* (Forward 5’-gccagggagttcaaggaca-3’, Reverse 5’-ctcgctgatgaatccgtcca-3’) primers were designed based on the barley predicted *F3’h* sequence AK363912 [39] found in NCBI database using BLASTp algorithm with *Zea mays* *F3’h* gene sequence HQ699781 [40] as a query (S1 File). *Ans* (Forward 5’-aagagggagtgggaggacta-3’, Reverse 5’-cgagggagaggatggcga-3’) primers were designed based on the contigs identified in the BARLEX database using wheat *Ans* sequence AB247918 as a query (S2 File). A fragment of the *Actin* gene sequence was used for reference purposes [41]. The amplifications were performed in an ABI Prism 7000 Sequence Detection System (Applied Biosystems, http://www.lifetechnologies.com) applying a SYNTOL SYBR Green I kit (http://www.syntol.ru/productmix.htm). Pre-determined amounts of cloned cDNA were used to generate standard curves. Each sample was run in three replicates. The differences among the lines were tested by Mann-Whitney *U*-test, taking *p*≤0.05 as significant.

### Chromatographic analysis of anthocyanins and related phenylpropanoids

Mature seeds were pulverized in a Retsch mill (Haan, Germany) and aliquots of 180 mg were extracted using 900 *μ*l of 70% MeOH with 2% formic acid. After centrifugation (28,000 *g* at 4°C for 10 min), the pellet was re-extracted with 600 *μ*l extraction solvent and centrifuged. Supernatants were combined and reduced under vacuum centrifugation to a final volume of 500 *μ*l. Then 200 *μ*l of extract were mixed with eluent A (see below) and stored at 4°C until analysis in the dark. Prior to chromatography, extracts were centrifuged again and filtered (Ultrafree MC-HV, PVDF, 0.45 *μ*m; Merck-Millipore, Darmstadt, Germany). The supernatant was filled into vials and 5 *μ*l were injected. Two independent extraction procedures were performed for each genotype studied.

Profiles of phenylpropanoids were obtained by using ultra-performance liquid chromatography (UPLC) in combination with photodiode array (PDA) as well as MS detection of separated compounds essentially with the system described by Petridis et al. [42] without recording fluorescent traces. Diode array data were extracted at 280 nm for phenolic compounds in general and at 515 nm for anthocyanins. The gradient was modified as follows: starting concentration was 98% eluent A (0.1% v/v formic acid in MS grade water) and 2% eluent B (0.1% v/v formic acid in acetonitrile, MS grade). 10% B were reached after 3 min, 20% B after 7 min and 98% B after 10 min in a linear mode. The column was flushed for further 3 min at this concentration and then equilibrated at the initial conditions prior to injection of the next sample. Anthocyanins were quantified using a calibration curve obtained by injecting cyanidin 3-*O*-glucoside as a standard at different concentrations with the help of the diode array data. A mass range from 50 to 1300 m/z was recorded.

## Results

### Microsatellite genotyping of the barley NILs

**Fig 2:**
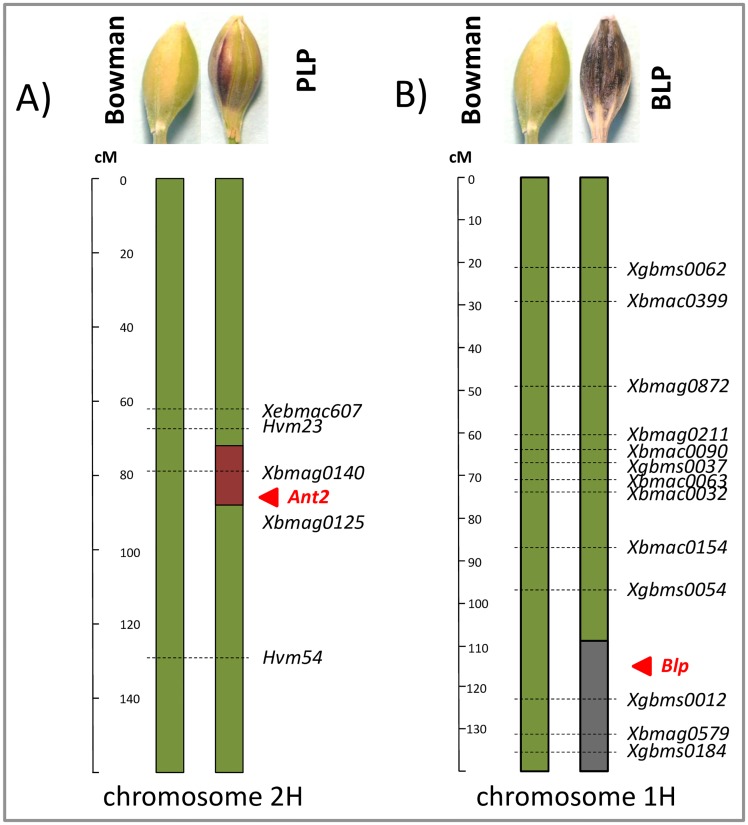
Microsatellite genotyping of the PLP (A) and BLP (B) lines. Purple and black colors mark introgressed fragments in the PLP and BLP lines, respectively.

Microsatellite genotyping of the barley NILs showed, that the PLP line has donor fragment located on chromosome 2HL, in the region of the microsatellite locus *Xbmag140*, near the *Ant2* locus, predetermining purple lemma and pericarp (Fig 2A).

In the BLP line, donor segment was located between microsatellites *Xgbms0012* and *Xgbms0184*, near the *Blp* gene, controlling black lemma and pericarp trait (Fig 2B). Thus, the NILs carry donor fragments with the genes, predetermining different colors of the barley lemma and pericarp.

### Structural and functional comparison of the different *Ant2* alleles

The nucleotide sequences of the *Ant2* genes of Bowman and the PLP line were determined and compared. The full-length nucleotide sequence 6,419 bp in length for the Bowman *Ant2* gene was found in the BARLEX database (S3 File). The PLP *Ant2* nucleotide sequence 5,066 bp in length was obtained by sequencing overlapping PCR products (the sequence was submitted to NCBI GenBank under accession number KX035100). The coding sequences of Bowman and PLP *Ant2* genes are split into eight exons with total length 1,680 bp (S2 Fig).

The *Ant2* nucleotide sequence identity between Bowman and PLP was 98% (the full-length exonic-intronic sequences were compared). The observed polymorphism between Bowman and PLP *Ant2* alleles (7 synonymous and 6 non-synonymous substitutions in the coding region and 45 SNPs and 2 indels 4, 5 bp in length within the intronic region) suggested the PLP to be a carrier of the *Ant2* allele from a donor parent (S2 Fig).

The predicted polypeptide sequence is 559 amino acid residues. Comparison with the known transcription factor LC, regulating anthocyanin biosynthesis in maize [43] revealed the conservative basic helix-loop-helix (bHLH) domain important for DNA binding and protein-protein interaction (S3 Fig). The observed non-synonymous substitutions between Bowman and PLP lay outside of the bHLH domain and are assumed to have no essential effect on the functionality of ANT2 since these substitutions are very similar by the level of hydrophobicity (S3 Table) and not changed the predicted secondary structure of the proteins (S4 Fig).

The *Ant2* genes of Bowman and PLP differ significantly in the 5’ regulatory region. In Bowman, a 179 bp insertion was identified in comparison with the PLP *Ant2* gene (S5 Fig). The insertion disturbs the order and spatial localization of the *cis*-acting regulatory elements identified in promotor regions of the genes. Among the identified elements those related to different stimuli such as light, biotic and abiotic stress factors, phytohormons and elements recognized by other regulatory factors participating in physiological functions have been identified (S4 Table).

**Fig 3:**
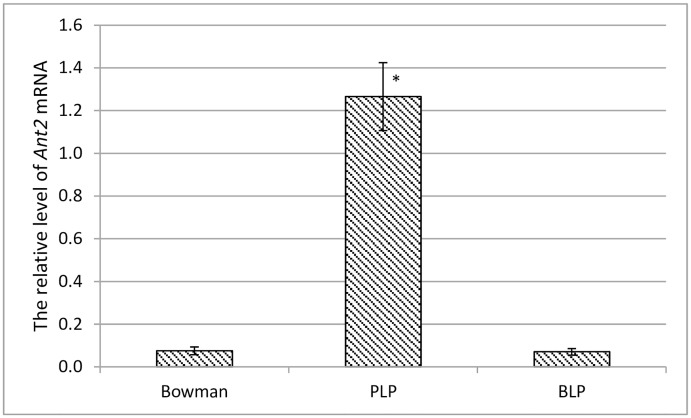
Expression of the *Ant2* gene in lemma and pericarp of NILs differing by the coloration. The data are presented as mean value ± standard error. *—differences are statistically significant between NILs and Bowman at *p* ≤ 0.05 (*U*-test).

The expression assay of the *Ant2* gene in grain of Bowman and the PLP line shows that level of the *Ant2* gene transcription was almost 17-fold abundant in the PLP line in comparison with Bowman and the BLP NIL (Fig 3). The data suggested that the rearrangements in promotor region of the *Ant2* gene lead to reduction of transcription level of *Ant2* in Bowman, resulting in the uncolored grain phenotype.

### Expression of the flavonoid biosynthesis structural genes in lemma and pericarp of differently colored barley grain

**Fig 4:**
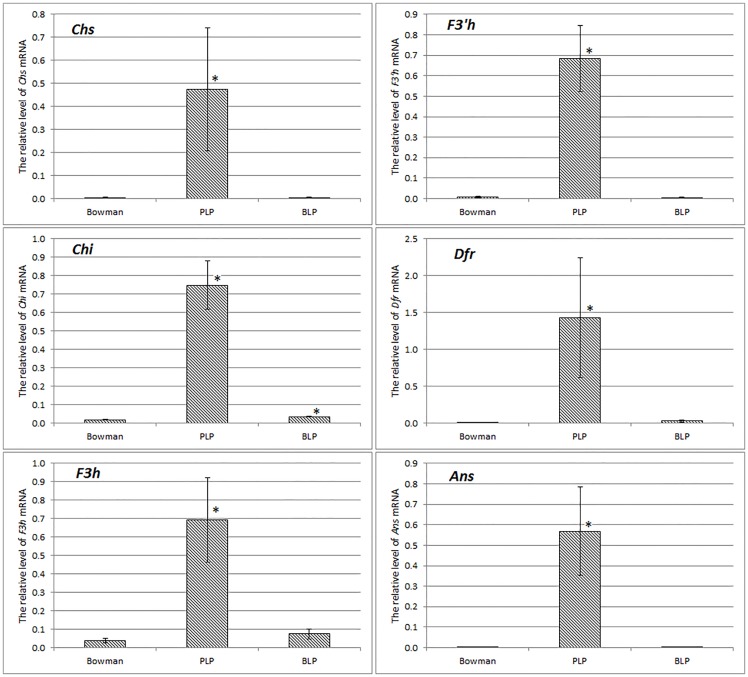
Expression of the flavonoid biosynthesis structural genes in grains of the barley NILs having different coloration of lemma and pericarp. The data are presented as mean value ± standard error. *—differences are statistically significant between NILs and Bowman at *p*≤ 0.05 (*U*-test).

To show regulatory functions of the *Ant2* gene and to investigate the involvement of the flavonoid pathway in black lemma and pericarp trait development the expression analysis of the flavonoid biosynthesis structural genes was performed in NILs having green, purple and black color of lemma and pericarp. In the purple-grained PLP line, expression of the structural genes *Chs*, *Chi*, *F3h*, *F3’h*, *Dfr*, and *Ans* was up-regulated in comparison with the green and black grains (Fig 4). All the genes were co-regulated as one regulatory unit. The data proved the regulatory functions of the *Ant2* gene in anthocyanin biosynthesis in barley grain pericarp.

In the black-grained BLP line, no differentially expressed genes (with the exception of *Chi*) in comparison with Bowman were found (Fig 4). The data demonstrated that there is no specific transcriptional regulation of the flavonoid biosynthesis in the black-grained barley line, suggesting that flavonoid pigments are not involved in development of black lemma and pericarp trait. Thus, distinct genetic networks underlay black and purple pigmentation of barley grain.

### Profiles of anthocyanins and related phenylpropanoids

**Fig 5:**
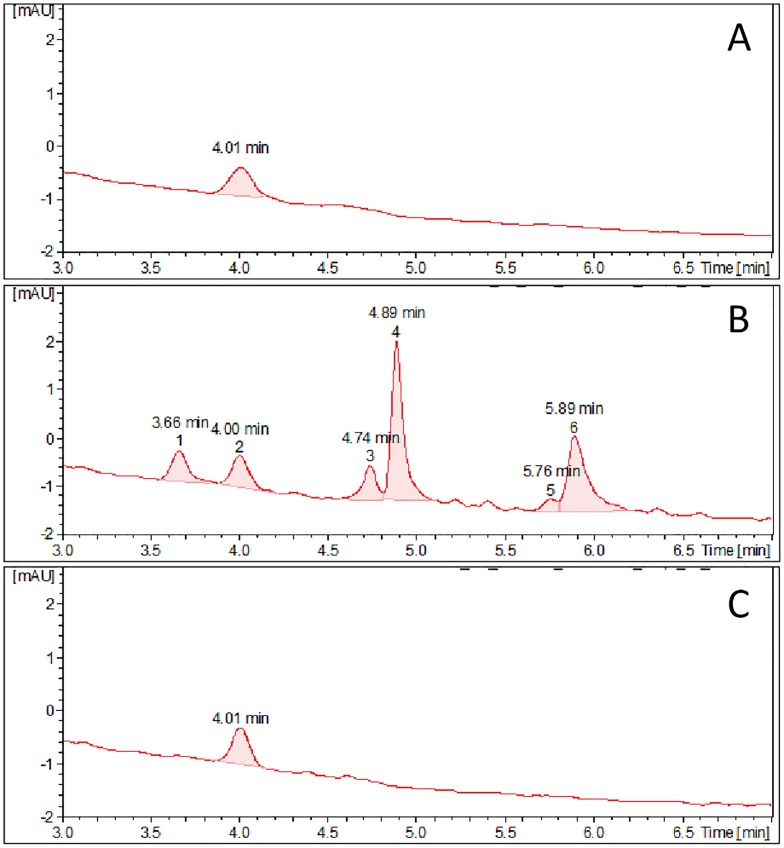
Anthocyanin profiles of Bowman (A), PLP (B) and BLP (C) genotypes. Seed extracts were prepared using acidified aqueous methanol as described in the materials and methods section. Extracts were separated by UPLC and compound elution was monitored by photodiode array (PDA) detection followed by MS analysis. The chromatograms were obtained by extracting the PDA data at 515 nm. X-axis represents time (min) and Y-axis represents absorbance in milliabsorbance units (mAU).

In order to correlate expression of the flavonoid biosynthesis pathway genes with metabolic profiles, methanolic whole seed extracts were separated by UPLC and compounds were detected by absorption as well as by mass spectrometry. Six peaks differing in elution time and mass could be detected in anthocyanin profile for the PLP genotype (Fig 5). In seed extracts of Bowman and BLP, only one compound corresponding in mass and elution time to peak two of PLP was found. Calculation of anthocyanin contents based on total peak areas showed similar low amounts in Bowman and BLP and about nine times higher value for anthocyanin content in the PLP line (S5 Table), consistent with the gene expression data (Figs 3 and 4).

Analysis of the PDA data at 280 nm revealed complex metabolic profiles of the three genotypes, representing phenolic compounds in general, but not excluding other cellular compounds with similar polarity (S6 Fig). Although the metabolic patterns of all three genotypes appeared similar, genotype BLP showed higher abundance in many of the individual compounds detected at 280 nm.

## Discussion

### New polymorphism underlies different grain color phenotype

In the current study, new polymorphism underlying different grain coloration in barley was revealed. The polymorphism is suggested to be associated with the rearrangement in the 5’ regulatory region of the *Ant2* gene in white-grained cultivar Bowman in comparison with its purple-grained NIL PLP. The rearrangement leads to reduction of the transcription level of the *Ant2* genes in Bowman (Fig 3) resulting in a decline of the anthocyanin biosynthesis structural genes expressions (Fig 4), absence of anthocyanins (Fig 5) and hence conferring a white-grained phenotype (S1 Fig).

Earlier the diagnostic marker distinguishing dominant and recessive alleles of the *Ant2* genes (*Ant2* / *ant2*) has been developed. The marker has been designed to a region carrying a 16-bp deletion within exon 6 that results in truncation of the predicted protein upstream of the bHLH domain [19]. Although the deletion has been identified in all 21 white-grained genotypes studied by Cockram et al. [19] and perfectly co-segregated with *ant2* alleles in biparental mapping population, it appeared to be not common for further white-grained barley cultivars, such as Bowman (S2 Fig). Also the *Ant2* marker was monomorphic between purple-grained Yuyaohongdamai and white-grained ACCA [24]. The authors [24] have been focused on the fine mapping of the *Pre2* gene which controls purple lemma and pericarp trait [23]. They have found close linkage (0.1 cM) of *Pre2* with the *HvOs04g47170* marker linked to *Ant2* allowed suggesting that *Pre2* is synonymous to *Ant2*, but because the *Ant2* marker has revealed no differences between the cultivars having different colors of grain the authors have stated that the gene controlling purple color in grain of Yuyaohongdamai is not *Ant2*. The transcriptional activity assay of the *Ant2* gene in purple-grained variety Retriever and white-grained variety Saffron has shown that the gene is transcribed in auricles, awns, and lemma of both cultivars; moreover transcriptional level of the *Ant2* gene did not differ between the cultivars [19].

Such different results on transcriptional activity of the *Ant2* gene in white-grained *vs* purple-grained lines/cultivars obtained in the current study (Fig 3) and by Cockram et al. [19] can be explained by different mutations in the *Ant2* gene leading to the white-grained phenotype: Cockram et al. [19] have revealed mutation causing structural damage of the ANT2 protein, but it did not influence the transcription of the gene; our data demonstrate another mutation which caused damage in transcriptional regulation of the *Ant2* gene but did not have influence on the structure of the protein.

Overall, the data on co-localization of the *Ant2* and *Pre2* genes, the absence of another bHLH-type gene in the region of localization of the *Ant2* gene [19, 24] and the differences in functional activity of the *Ant2* gene in colored and uncolored barley grains (Fig 3) suggest together that purple color of barley grain is controlled by *Ant2* that is synonymous to the *Pre2* gene.

### Co-regulation of the anthocyanin biosynthesis pathway genes in barley grain

Although the molecular-genetic basis for anthocyanin biosynthesis is common for the plant species: structural genes encoding for enzymes of the pathway (Fig 1) are regulated by transcriptional factors of MYB-, bHLH- (MYC-) and WD40–types [44], some species-specific differences have been noted in the regulation of the structural genes [45, 46]. Depending on co-expression of the structural genes in the presence of the dominant alleles of the regulatory genes following patterns of the anthocyanin biosynthesis regulation could be distinguished: (1) the set of the genes is co-regulated separately from the others genes (the subsets of the ‘early’ and ‘late’ genes); (2) anthocyanin biosynthesis is regulated at a certain stage of the pathway; (3) whole set of the genes is co-regulated as one regulatory unit.

The first regulatory pattern, for example, has been observed in petals of *Petunia hybrida* where the ‘late’ genes *Dfr*, *Ans*, *Mt*, and *Rt* were co-regulated by transcriptional factors AN1, AN2, and AN11 (bHLH, MYB, and WD40, respectively) separately from the ‘early’ genes *Chs*, *Chi*, and *F3h* [45]. In flowers of *Antirrhinum majus*, the ‘late’ genes *F3h*, *Dfr*, *Ans*, and *Ufgt* where co-expressed in the presence of DELILA (bHLH type) regulatory factor separately from the ‘early’ genes *Chs*, *Chi* [47]. In seedlings of *Oryza sativa*, *F3h*, *Dfr*, and *Ans* were co-regulated by the OSB2 (bHLH-type) transcriptional regulator separately from *Chs*, *Chi*, *F3’h* [48, 49].

The second pattern of the anthocyanin pathway regulation is similar to the first one, but characterized by specific regulation of one of the structural genes in dependence of the dominant allele of the regulatory genes. Such regulation pattern was observed in berries of *Vitis vinifera* [50, 51] and pericarp of *Litchi chinesis* fruits [52, 53], where anthocyanin biosynthesis is regulated at a stage of the *Ufgt* gene expression. In grain pericarp of *Triticum aestivum*, the pathway is regulated at a step of *F3h* gene expression [54].

The third regulatory pattern has been revealed in aleurone layer of kernel and husk of *Zea mays*, where *Chs*, *Dfr*, and *Ufgt* were co-regulated in the presence of the dominant allele *R*(*S*)/*C1* and *B*/*Pl* (MYB and bHLH, respectively) [55, 56]. As shown in the current study, the anthocyanin biosynthesis structural genes in barley grain are co-regulated as one regulatory unit in the presence of the ANT2 (bHLH) regulatory factor. Similar phenomenon has been observed by Meldgaard M. [12], who has studied transcription of the *Chs*, *F3h*, and *Dfr* genes in testa pericarp tissues in the proanthocyanidin-free barley *ant13* mutant line. This regulatory pattern is characteristic not only to such monocot plant species as maize and barley. The whole set of the genes is co-expressed in dicot plants having parts colored by anthocyanins such as flash of tuberous roots in *Ipomoea batatas* [57], skin of apple fruits [58], or taproots in *Daucus carota* [59, 60].

As seen the regulation pattern of anthocyanin biosynthesis in barley grain is not unique in comparison with the other plant species but it is quite different from such cereal relatives as wheat and rice.

### ‘Pure’ melanin-like pigmentation of the black grains of barley

Black color of barley lemma and pericarp is caused by melanin-like pigments, called phytomelanins [2]. Plant ‘Melanins’ is a general name for groups of high-molecular black/brown pigmented irregular polymers, arising in the course of oxidation and polymerization of phenolic compounds. Although black pigmentation is considered to be an important agronomic trait because of its protective functions against severe environments and infections [61–63] chemical nature of the black pigments and the biosynthesis pathway leading to them is not clear [61, 64].

Some studies of phytochemical composition of barley grains with black color have revealed that the real melanin-like black pigments can be mixed with anthocyanins and other related copigments that contribute to the total content of phenols [65, 66] and prevent determination of ‘pure’ melanin-like compounds and their precursors in barley.

In the current study, the comparative chromatographic analysis of whole seed extracts and transcriptional assay of the flavonoid biosynthesis pathway genes was performed for the NILs having different colors of pericarp and lemma (S1 Fig). Although the minor peak corresponding to the anthocyanins was detected in whole seed extracts of black-grained line as well as of uncolored Bowman (Fig 5), none of the studied genes involved in flavonoid biosynthesis is expressed significantly in lemma and pericarp of these genotypes (Figs 3 and 4). This allows suggesting that anthocyanins and the other flavonoids unlikely participate in black pigmentation of barley lemma and pericarp and identified peak may belong to compound from the others parts of grain such as seed coat, aleurone layer or endosperm. The lines studied have similar phenylpropanoid profiles with higher abundance in many of the individual compounds in BLP (S6 Fig). Further studies are needed to address the question about the chemical composition of black barley grains and metabolic pathway leading to them. The characterized NILs are proper model for this goal.

## Supporting Information

S1 Fig Spikes and grains of Bowman (A), PLP (B) and BLP (C) lines used in the current study. (DOCX)

S2 Fig Alignment of the *Ant2* gene nucleotide sequences of Bowman and PLP line. Exonic sequences are marked by green color.
(DOCX)

S3 Fig Alignment of the ANT2 proteins of Bowman and PLP and the related LC protein, regulating anthocyanin biosynthesis in maize. Conservative basic helix-loop-helix (bHLH) domain is marked.
(DOCX)

S4 Fig Secondary structure of the ANT2 protein of Bowman and PLP predicted by CFSSP Server. The residues differing in Bowman and PLP are marked by pink color.
(DOCX)

S5 Fig Alignment of the nucleotide sequences of the 5’ regulatory region of the *Ant2* gene of Bowman and PLP line. Exonic sequences are marked by green color.
(DOCX)

S6 Fig Phenylpropanoid profiles of Bawman, PLP and BLP seed extracts. The overlay of chromatograms obtained by extracting the PDA data at 280 from extract 1 of all genotypes is shown. Chromatograms are depicted in black (Bowman), blue (PLP) and red (BLP).
(DOCX)

S1 Table Data on the structural and regulatory genes for flavonoid biosynthesis in barley identified up to date. (DOCX)

S2 Table Primer pairs amplifying *Ant2* gene 5’ regulatory region designed in the current study. (DOCX)

S3 Table Amino acid substitutions identified between ANT2 of Bowman and PLP and its classification based on the polarity of the side chain group. (DOCX)

S4 Table Putative *cis*-acting regulatory elements identified in promoter regions of Bowman (936 bp before ATG codon) and PLP (754 bp before ATG codon) *Ant2* genes using the PLACE database. (DOCX)

S5 Table Quantification of total anthocyanins in grains of Bowman, PLP and BLP. Peaks areas of PDA data at 515 nm were integrated. The sum of all peak areas was taken for calculating total anthocyanin contents. A calibration curve using authentic cyanidin 3-*O*-glucoside was used.
(DOCX)

S1 File The nucleotide sequences of Morex, Barke, and Bowman contigs corresponding to the *F3’h* gene found in BARLEX database. (DOCX)

S2 File The nucleotide sequences of Bowman contigs corresponding to the *Ans* gene found in BARLEX database. **Reconstructed full-length nucleotide sequence of *Ans* is also present**. Yellow color marks coding sequence of the gene.
(DOCX)

S3 File The nucleotide sequence of Bowman contig corresponding to the *Ant2* gene found in BARLEX database. Exonic sequences are marked by green color.
(DOCX)
