# Supplementary figures and images for: Regulation of the Flavonoid Biosynthesis Pathway Genes in Purple and Black Grains of Hordeum vulgare
Source: PLoS One. 2016 Oct 5;11(10):e0163782. doi: 10.1371/journal.pone.0163782 (PMC5051897; doi:10.1371/journal.pone.0163782)

**S1 Fig. Spikes and grains of Bowman (A), PLP (B) and BLP (C) lines used in the current study.**


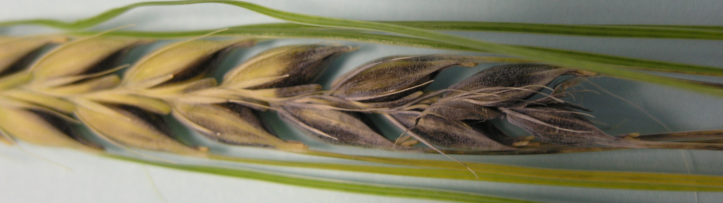

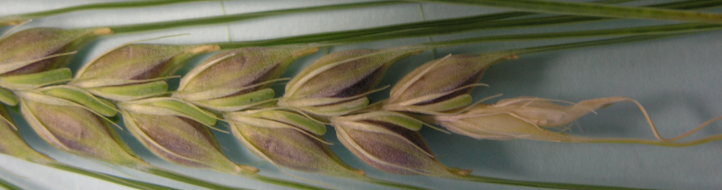

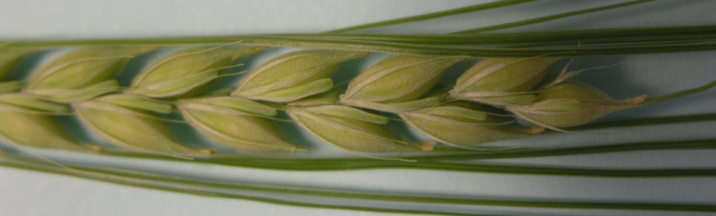

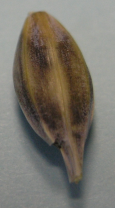

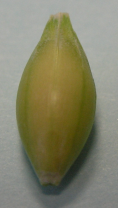

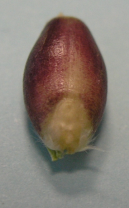

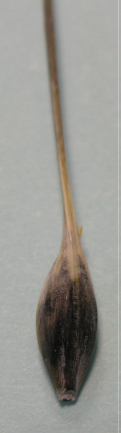

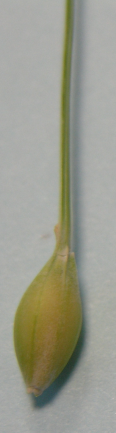

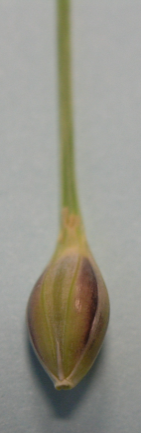


**C**

**B**

**A**

**C**

**C**

**B**

**B**

**A**

**A**

Supplement: S1 Fig — (DOCX) [file pone.0163782.s001.docx]
