# Supplementary material for: Regulation of the Flavonoid Biosynthesis Pathway Genes in Purple and Black Grains of Hordeum vulgare
Source: PLoS One. 2016 Oct 5;11(10):e0163782. doi: 10.1371/journal.pone.0163782 (PMC5051897; doi:10.1371/journal.pone.0163782)
Supplement: S2 Fig — Exonic sequences are marked by green color. (DOCX) [file pone.0163782.s002.docx]

**S2 Fig. Alignment of the *Ant2* gene nucleotide sequences of Bowman and PLP line.** Exonic sequences are marked by green color.

exon 1

**Bowman ATGGCGCTACCAATAGTTCGTCCGAGCCAGGAAGAACCGCCGACGGGGAAGCAATTCAGCTACCAGCTCGCTGCCGCTGTGAGGAGCATCAACTGGAGCTACGCCATATTCTGGTCCATTTCAAC**

**PLP ATGGCGCTACCAATAGTTCGTCCGAGCCAGGAAGAACCGCCGACGGGGAAGCAATTCAGCTACCAGCTCGCTGCCGCTGTGAGGAGCATCAACTGGAGCTACGCCATATTCTGGTCCATTTCAAC**

exon 2

**Bowman CAGCCGTCCAGGGTAGGGAGTGCATCAGATTGATGATCACTTGGCACTGGCCGTTTCCTTTCCTCTTATGATCCGTTTGTTATGTAGGGTACTGACCTGGAAGGACGGGTTCTACAACGGCGAGA**

**PLP CAGCCGTCCAGGGTAGGGAGTGCATCAGATTGATGATCACTTGGCACTGGCCGTTTCCTTTCCTCTTATGATCCGTTTGTTATGTAGGGTACTGACCTGGAAGGACGGGTTCTACAACGGCGAGA**

**Bowman TAAAGACGAGGAAGGTCACCAGCTCGGCGGACCTCACCGCCGACCAGCTCGTCCTGCAGAGGAGCGAGCAGCTGCGGGAGCTCTACCAGTCCCTCTTGTCCGGCCAGTGCGACCACCGGGGGAGG**

**PLP TAAAGACGAGGAAGGTCACCAGCTCGGCGGACCTCACCGCCGACCAGCTCCTCCTGCAGAGGAGCGAGCAGCTGCGGGAGCTCTACCAGTCCCTCCTGTCCGGCCAGTGCGACCACCGGGGGAGG**

**Bowman CGCCCGGCCGCCGCGCTCTCGCCGGAGGACCTCGGGGACGCCGAATGGTACTACGCCGTCTGCATGAGCTATGCCTTCCGCCCTGGCCAAGGGTATGTACTACTCCGTAGTATGCTTCAAGAACA**

**PLP CGCCCGGCCGCCGCGCTCTCGCCGGAGGACCTCGGGGACGCCGAATGGTACTACGCCGTCTGCATGAGCTATGCCTTCCGCCCTGGCCAAGGGTATGTACTACTCCGTAGTATGCTTCAAGAACA**

**Bowman ACTCCTCTGATCCACAA GATAACA TTTATCAATAGTTAACAATCATCTTATCTTGATAAGATGAGAGTTATTTTGCTAGAGAGTGAAAACTACAACGTTTGCTATTTCGTCATCAG**

**PLP ACTCCACTGATCCATAACGGCCGATGCCTGCGCACCTACAGTAGTTAGCAATCATCTTATCTTGATAAGATAAGATTTACTTTGCTAGAGAGTGAAAACTACAACGTTTGCTATTTCGTCATCAG**

exon 3

**Bowman GTTGCCAGGCAGAAGCTTTGCGAGCAACGAGCCTGTTTGGCTGTGCAATGCTCAGTGCGCAGACACCAAAACTTTCCAACGCTCGCTCTTAGCGAAGGTACGTTTGATTGCACCGCCCGAGTGTC**

**PLP GTTGCCAGGCAGAAGCTTTGCGAGCAACGAGCCTGTTTGGCTGTGCAATGCTCAGTGCGCAGACACCAAAACTTTCCAGCGCTCGCTCTTAGCGAAGGTACGTTTGATTGCACCGCCCGAGTGTC**

**Bowman TCGCAAGCCTCACTCTCATGTAGTCCCTCCGTAAATAAATGGAGTATAAGAGTGTTTAGATCACTACTTAGTGATCTAAACGCTTTTATGTTTCTTTACGAAGGGAGTACTATCTGCCTAATATG**

**PLP TCGCAAGCCTCACTCTCATGTAGTCCCTCCGTAAATAAATGGAGTATAAGAGTGCTTAGATCACTACTTAGTGATATAAACGCTTTTATGTTCCTTTACGAAGGGAGTACTATCTGCCTAATATG**

exon 4

**Bowman TACCCGATCTTGACTAATACCATGTTGTCTACTGTGTACTTTTGGCGCACATACTATATCTGTCGGCTAAATCAGACGACGTCTATCCAGGTTAGCTAGCGCATGTCATGCACCCTATATCTGTG**

**PLP TACCCGATCTTGACTAATACCATGTTGTCTACTGTGTACTTTTGGCGCACATACTATATCTGTCGGCTAAATCAGACGACGTCTATCCAGGTTAGCTAGCGCATGTCATGCACCCTATATCTGTG**

exon 5

**Bowman TATACTGTTCATGTTTCTGACCCCAGCTGCACGGGTGACAATTTTTTTTCTTCAGACGGTCGCCTGCATTCCCTTGATGGGTGGTGTGCTTGAGCTCGGGACGACAGATACCGTGAGCTTTCCTC**

**PLP TATACTGTTCATGTTTCTGACCCCAGCTGCACGGGTGACAATATTTTTTCTTCAGACGGTCGCCTGCATTCCCTTGATGGGTGGTGTGCTTGAGCTCGGGACGACAGATACCGTGAGTTTTCCTC**

**Bowman GCATCAAGCACATGATATTCACTTACGGTTTAAAATTAAACGGAAAACTCACTATTAGTAACGCTTTGTCGCATCAAGCACATCGAAATTGCAAAATTTAACCCGGGTTCCATGGAGCCTTGTTG**

**PLP GCATCAAGCACATGATATTCACTTACGGTTTAAAATTAAACGGAAAACTCACTATTAGTAACGCTTTGTCTCATCAAGCACATCGAAATTGCAAAATTTAACCCGGGTTCCATGGAGCCTTGTTG**

**Bowman TTAAGAAAAACACAACAGAAGTTGTGAGCACCTATATATTTCCGTTCAGTACATCTTGCCATATGTGGG TAGACAACAAAGACAAATATGAGGATCTACAAATAGCCAATAATAGATGTGCTA**

**PLP TTAAGAAAAACACAACAGAAGTTGTGAGCACCTATATATTTCCGTTCAGTACATCTTGCCATATGTGGGGGTAGACAACAAAGACAAATATGCGGATCTACAAATAGCCAATAATAGATGTGCTA**

**Bowman GTATTTCCAAAACTACACATTGTCATCTTGTGTAGCTCACATGCATGTGTTATAGTGTTTATTATCGAATGCTACGTATATGCTAGTATAGATCTATATATACATCTAGGTAGACTTGTTTTACT**

**PLP GTATTTCCAAAACCACACATTGTCATCTTGTGTAGCTCACATGCATGTGTTATAGTGTTTATTATCGAATGCTACGTGTATGCTAGTATAGATCTATATATACATCTAGGTAGACTTGTTTTACT**

**Bowman GTTTTTTTTCAAAGTTGTAAATATTATTTGATTTTTTTTCCAAAATTTCAGTCTCACTACAACCCTGATCTCCCAATTATTCGCTCAAGCTGCCATGTCAACTATGGTACAAGTTTGAAGTTTGT**

**PLP GTTTTTTTTCAAAGTTGTAAATATTATTTGATTTTTTTTCCAAAATTTCAGTCTCACTACAACCCTGATCTCCCAATTATTCGCTCAAGCTGCCATGTCAACTATGGTACAAGTTTGAAGTTTGT**

**Bowman TACCCTTTCATTCTTTCCCTGCCCTTGTTGCTTACAAATGCGAGCTTTGCTACAAGTACGGACGTTTTACAGGATTTTTACAGGCAAAGTTGAAGTGGCTTATTTTAATTGATACTTAGATGAGG**

**PLP TACCCTTTCATTCTTTCCCTGCCCTTGTTGCTTACAAATGCGAGCTTTGCTACAAGTACAGACGTTTTACAGGATTTTTACAGGCAAAGTTGAAGTGGCTTATTTTAATTGGTACTTAGATGAGG**

**Bowman CCGGCCCTAAAAATCAAGGGGAGATTAGTGAGGAGAAAAATCCATCAGTCTGTAACCTTCTGTAAAAAAAAGGTCCGTAGGTCTGGCATTTTCCTA CAAATGCGGACGAAACCAATCTCTTTTG**

**PLP CCGGCCCTAAGAATCAAGGGGAGATTAGTGAGGAGAAAAACCCATCAGTCTGTAATCTTTTGTAAAAAA GGTCCGTAGGTCTGGCATTTTCCTATAAAATGCGGACGAAACCAATATCTTTTG**

**Bowman AGGCCTCTGCTACGTTATTCTAGTGTGTTATTTTTCAAGAACAAGAAACACAATGCTGCATGTCATTGTCATTAAGAAGGAAAGGGAGTTAATTAGAAGCAAATAGTCGTGACAAAGCATGTAGT**

**PLP AGGCCTCTGCTACGTTATTCTAGTGTGTTATTTTTCAAGAACAAGAAACACAATGCTGCATGTCATTGTCATTAAGAAGGAAAGGGAGTTAATTACAAGCAAATAGTCGTGACAAAGCATGTAGT**

**S1 Fig.** *Cont.*

**Bowman GTTGTTATACCACACGTGAGACCACCGCCACCACATTCATATCTAGGCCCACTCCTCCCTAATCTTTGTCACTGACTGCCTAGCCTTTTTGGAAAGCATAAGTTCTTGTGCCGCCGGTGCGACCA**

**PLP GTTGTTATACCACACGTGAGACCACCGCCACCACATTCATATCTAGGCCCACTCCTCCCTAATCTTTGTCACTGACTGCCTAGCCTTTTTGGAAAGCATAAGTTCTTGTGCCGCCGGTGCGACCA**

**Bowman CTCTTTGCACCGATGACACTTTCCCATCAAAAACTCTAGTGTTGCGCTCTAGCCTTCATTAATGTCAGCCAGCCGGTCTTGAACGACAATGAAGTCTTCCAAGTCGCCCACAACGTGAGGTTGCA**

**PLP CTCTTTGCACCGATGACACTTTCCCATCAAAAACTCTAGTGTTGCGCTCTAGCCTTCATTAATGTCAGTCAGCCGGTCTTGAACGACAATGAAGTCTTCCAAGTCGCCCACAACGTGAGGTTGCA**

**Bowman AATCTACTTTGGTGAAGGCACATGTGCGTATAGCCCCAAATACGTGCACAGAAATTTGGCCACTTGATTTGCAAGAGAAGCGGTCAGAACATCCAGACAATCCACTCCCTCGCATCGGGTAGGTG**

**PLP AATCTACTTTGGTGAAGGCACATGTGCGTATAGCCCCAAATACGTGCACAGAAATTTGGCCACTTGATTTGCAAGAGAAGCGGTCAGAACATCCAAACAATCCACTCCCTCGCATCGGGTAGGTG**

**Bowman AAATCACATGTTTCTAAAGATTAACCACAAGACCAAAATCGCCACCAAATAACGTCAAAGATCTCCTTCCTTGCCAACAACTCGCTTTCTCTGGGTTTGAGGTAACCCGATAGCCGGTGACGAAC**

**PLP AAATCACATGTTTCTAAAGATTAACCACAAGACCAAAATCGCCACCAAATAACGTCAAAGATCTCCTTCCTTGCCAACAACTCGCTTTCTCTGGGTTTGAGGTAACCCGATAGCCGGTGACGAAC**

**Bowman ACCCCATCTATCCAAAGGTTCAGACACACCAAAATCCTCCACTTTATTCCTTGTGCAGGTGGTAAAGCCCCTTGCCTCGTGGCATGACAATAGGTTGTCCTGATTTTGTTTCTCGGATTCGTCAT**

**PLP ACCCCATCTATCCAAAGGTTCAGACACACCAAAATCCTCCACTTTATTCCTTGTGCAGGTGGTAAAGCCCCTTGCCTCGTGGCATGACAATAGGTTGTCCTGATTTTGTTTNTCGGATTCGTCAT**

**Bowman AAAGGGGAGGCTTTTATACCGTATGATTGAAAATTTTGTATTCTATCAGTAAATTACGATAAATAAATAAAAATACTTCTTTTAATGTAAAATATATTCTTATACTATGTGACAATGGGAAGGCT**

**PLP AAAGGGGAGGCTTTTATACCGTATGATTGAAAATTTTGTATTCTATCAGTAAATTACGATAAATAAATAAAAATACTTCTTTTAATGTAAAATATATTCTTATACTATGTGACAACGGGAAGGCG**

**Bowman GTACCGTTTGCTCGGGACACTCATTAAGTCATGAAGACACAATACTACTAAATCAATCCATGAATTGTTTGAGTTCTTCACCAAATTAAATGCTACTGTGCTGATTAATTCATTCGAATATAAGT**

**PLP GTACCGTTTGCTGGGGACACTCATTAAGTCATGAAGACACAATACTACTAAATCAATCCATGAATTGTTTGAGTTCTTCACCAAATAAAATGCTACTGTGCTGATTAATTCATTCGAATATAAGT**

exon 6

**Bowman TAAAAATTAACCAAAGCACTATCAATCGATTTGCTAGGTTTTGGAGGACAGAGACATGGTGAACCGAATCAGCACATCTTTCTGGGACCTGAAGATCCCAACAAGCTCGAAGCCGAAGGAGCCCT**

**PLP TAAAAATTAACCAAAGCGCTATCAATCGATTTGCTAGGTTTTGGAGGACAGGGACATGGTGAACCGGATCAGCACATCTTTCTGGGACCTGAAGATCCCAACAAGCTCGAAGCCGAAGGAGCCCT**

**Bowman CCAGCCCATCAGCAGACGACGCTGGTGAGGCCGACATCGTGTTCCAAGACCTCGACCACAACACCATGGCCGCGATGATCCCCGGGGAACTCGAGCTAGGGGAGGTCGAGTGCCTGTCCGACGAC**

**PLP CCAGCCCATCAGCAGACGACGCTGGTGAGGCCGACATCGTGTTCCAAGACCTCGACCACAACACCATGGCCGCGATGATCCCCGGGGAACTCGAGCTAGGGGAGGTCGAGTGCCTGTCCGACGAC**

**Bowman AACCTCGAGCGGATCACGAAGGAGATCAACGGGTTCTACGGCCTGTGCGACGAGCTGGACGTCGGCGCTCTCGACGAAAACTGGATCATAGGCGGGTCTTTCGAGGTCATGTCCTCGCCGGAAGC**

**PLP AACCTCGAGCGGATCACGAAGGAGATCAAACGGTTCTACGGCCTCTGCGACGAGCTGGACGTCGGCGCTCTCGACGAAAACTGGATCATAGGCGGGTCTTTCGAGGTCATGTCCTCGCCGGAAGC**

**Bowman GCCACCGGCGCCTGCAGCCACCGGCGGCATCACTGATGGTATTGTCACTTTAAGCGCCGCTGCGTCCTCTCTCTCATCGTGCTTTACGGCTTGGAAGAGATCGTGGGACTCAGCCGAAGACATGG**

**PLP GCCACCGGCGCCTGCAGCCACCGGCGGCATCACTGATGGTATTGTCACTTTAAGCGCCGCTGCGTCCTCTCTCTCATCGTGCTTTACGGCTTGGAAGAGATCGTGGGACTCAGCCGAAGACATGG**

**Bowman CTGCGCCGGTCGCCGGGCAGTCGCAGAAGTTACTGAAGAAAGCTTTGGCCGGTGGTGCGTGGGCGATTAACGGCGGCGGCGGCGGCGGCACGGCGAGAGCTCAGGAAAGTAGCAACACCAAGAAC**

**PLP CTGCGCCGGTCGCCGGGCAGTCGCAGAAGTTACTGAAGAAAGCTTTGGCCGGTGGTGTGTGGGCGATTAACGGCGGCGGCGGCGGCGGCACGGCGAGAGCTCAGGAAAGTAGCAACACCAAGAAC**

**Bowman CATGTCATTTCGGAGAGGCGGCGCCGGGAGAAGCTCAACGAGATGTTCCTGATTCTCAAGTCACTGGTGCCGTCCATTCACAAGGTAACACGCGCACATCACCGGAAAGCAAAAGAAAAA TACT**

**PLP CATGTCATATCGGAGAGGCGGCGCCGGGAGAAGCTCAACGAGATGTTCCTGATTCTCAAGTCACTGGTGCCGTCCATTCACAAGGTAACACGCGCACATCACCGGAAAGCAAAAGAAAAAATACT**

exon 7

**Bowman GTCCAGAGATGTGTCTCTGAACTTTTGTTTGCAGGTGGACAAGGCATCCATCCTAGCTGAGACGATAGCCTATCTCAGAGAGCTGGAGCAAAGGGTGGAGGAGCTAGAATCCAACAGGGCGCCGT**

**PLP TTCCAGAGATGTGTTTCTGAACTTTTGTTTGCAGGTGGACAAGGCATCCATCCTAGCTGAGACGATAGCCTATCTCAGAGAGCTGGAGCAAAGGGTGGAGGAGCTAGAATCCAACAGGGCGCCGT**

**Bowman CGCGGCCGGCCGGAGCAGCCGTCCGGAGACACCATGACGCCGCGGCGAAGAAGATGTTAGCTGGATCCAAGAGAAAGGCGTCGGAGCTCGGCGGGGACGACGGCCCGAACAGCGTCGTCAACGTC**

**PLP CGCGGCCGGCCGGAGCAGCCGTCCGGAGACACCATGACGCCGCGGCGAAGAAGATGTTAGCTGGATCCAAGAGAAAGGCGTCGGAGCTCGGCGGGGACGACGGCCCGAACAGCGTCGTCAACGTC**

**Bowman ACGGTGATGGAGAAAGAGGTGCTCCTGGAGGTGCAATGCCGGTGGAAGGAGCTGCTGATGACGCAGGTGTTCGACGCCATCAAGAGCCTCCGCCTGGACGTGCTCTCCGTGCGCGCGTCGACGCC**

**PLP ACGGTGACGGAGAAAGAGGTGCTCCTGGAGGTGCAATGCCGGTGGAAGGAGCTGCTGATGACGCAGGTGTTCGACGCCTTCAAGAGCCTCCGCCTGGACGTGCTCTCCGTGCGCGCGTCGACGCC**

**S1 Fig.** *Cont.*

**Bowman CGACGGCCTCCTCGCTCTCAAGATACGAGCTCAGGTCCGTCTGGTAGCAGCTGATTATTTAAGCTCCATCCGGCGGTAAAAA TGCAGCATTAATTAACGCTCGTATGCATGGCTATGGTTTACT**

**PLP CGACGGCCTCCTCGCTCTCAAGATACGAGCTCAGGTCCGTCTGGTAGCAGCTGATTAACTTAGCTCCATCCGGCGGTAAAAAATGCAGCATTAATTAACGCTCGTATGCATGGCTATGGTTTACT**

exon 8

**Bowman GTTGCAGTTCGCCGGTCCTGGCGCCGTGGAGCCCGGGATGATCATCGGAGCGCTTCAGACAGCTACACGAGGGCGCTGA**

**PLP GTTGCAGTTCGCCGGTCCTGGCGCCGTGGAGCCCGGGATGATCATCGGAGCGCTTCAGACAGCTACACGCGGGCGCTGA**
