# Supplementary material for: Regulation of the Flavonoid Biosynthesis Pathway Genes in Purple and Black Grains of Hordeum vulgare
Source: PLoS One. 2016 Oct 5;11(10):e0163782. doi: 10.1371/journal.pone.0163782 (PMC5051897; doi:10.1371/journal.pone.0163782)
Supplement: S3 Fig — Conservative basic helix-loop-helix (bHLH) domain is marked. (DOCX) [file pone.0163782.s003.docx]

**S3 Fig. Alignment of the ANT2 proteins of Bowman and PLP and the related LC protein, regulating anthocyanin biosynthesis in maize.** Conservative basic helix-loop-helix (bHLH) domain is marked.

**1 10 20 30 40 50 60 70 80**

**Bowman MALPIVRPSQEEPPTGKQFSYQLAAAVRSINWSYAIFWSISTSRPGVLTWKDGFYNGEIKTRKVTSSADLTADQLVLQRS**

**PLP MALPIVRPSQEEPPTGKQFSYQLAAAVRSINWSYAIFWSISTSRPGVLTWKDGFYNGEIKTRKVTSSADLTADQLLLQRS**

**Maize_LC (M26227) MALSASRVQQAE~AERQLMRSQLAAAARSINWSYALFWSISDTQPGVLTWTDGFYNGEVKTRKISNSVELTSDQLVMQRS**

**81 90 100 110 120 130 140 150 160**

**Bowman EQLRELYQSLLSGQCDHRGRRPAAALSPEDLGDAEWYYAVCMSYAFRPGQGLPGRSFASNEPVWLCNAQCADTKTFQRSL**

**PLP EQLRELYQSLLSGQCDHRGRRPAAALSPEDLGDAEWYYAVCMSYAFRPGQGLPGRSFASNEPVWLCNAQCADTKTFQRSL**

**Maize_LC (M26227) DQLRELYEALLSGEGDRRA~RPAGSLSPEDLGDTEWYYVVSMTYAFRPGQGLPGRSFASDEHVWLCNAHLAGSKAFPRAL**

**161 170 180 190 200 210 220 230 240**

**Bowman LAKTTSIQTVACIPLMGGVLELGTTDTVLEDRDMVNRISTSFWDLKIPTSSKPKEPSSPSADDAGEADIVFQDLDHNTMA**

**PLP LAKTTSIQTVACIPLMGGVLELGTTDTVLEDRDMVNRISTSFWDLKIPTSSKPKEPSSPSADDAGEADIVFQDLDHNTMA**

**Maize_LC (M26227) LAKSASIQSILCIPVMGGVLELGTTDTVPEAPDLVSRATAAFWEPQCPSSSPSGRANETGEAAADDGTFAFEELDHN~IE**

**241 250 260 270 280 290 300 310 320**

**Bowman AMIPGELELGEVECLSDDNLERITKEINGFYGLCDELDVGALDENWIIGGSFEVMSSPEAPPAPAATGGITDGIVTLSAA**

**PLP AMIPGELELGEVECLSDDNLERITKEIKRFYGLCDELDVGALDENWIIGGSFEVMSSPEAPPAPAATGGITDGIVTLSAA**

**Maize_LC (M26227) AMTA~ELRLREAEALSDD~LEHITKEIEEFYSLCDEMDLQAL~DGWTVDAS~EVPCSSPQPAPPPVDRATANVAADASRA**

**321 330 340 350 360 370 380 390 400**

**Bowman ASSLSSCFTAWKRSWDSAEDMAAPVAGQSQKLLKKALAGGAWAINGGGGGGTARAQESSNTKNHVISERRRREKLNEMFL**

**PLP ASSLSSCFTAWKRSWDSAEDMAAPVAGQSQKLLKKALAGGVWAINGGGGGGTARAQESSNTKNHVISERRRREKLNEMFL**

**Maize_LC (M26227) P~SRATSFMAWTRS~SSCSDDAAPAA~EPQRLLKKVVAGGGAWESCGGATGAAQEMSGTGTKNHVMSERKRREKLNEMFL**

basic

Helix 1

**401 410 420 430 440 450 460 470 480**

**Bowman ILKSLVPSIHKVDKASILAETIAYLRELEQRVEELESNRAPSRPAGAAVRRHHDAAAKKMLAGSKRKASELGGDDGPNSV**

**PLP ILKSLVPSIHKVDKASILAETIAYLRELEQRVEELESNRAPSRPAGAAVRRHHDAAAKKMLAGSKRKASELGGDDGPNSV**

**Maize_LC (M26227) VLKSLLPSIHRVNKASILAETIAYLKELQRRVQELESSREP~RPSETTTRL~NESVRKEVCAGSKRKSPELGRDD~GTSN**

Helix 2

Loop

Helix 1

**481 490 500 510 520 530 540 550 559**

**Bowman VNVTVMEKEVLLEVQCRWKELLMTQVFDAIKSLRLDVLSVRASTPDGLLALKIRAQFAGPGAVEPGMIIGALQTATRGR**

**PLP VNVTVTEKEVLLEVQCRWKELLMTQVFDAFKSLRLDVLSVRASTPDGLLALKIRAQFAGPGAVEPGMIIGALQTATRGR**

**Maize_LC (M26227) VTVTVSDKDVLLEVQCRWEELLMTRVFDAIKSLHLDVLSVQASAPDGFMGLKIRAQFAGSGAVVPWMISEALRKAIGKR**
