# Supplementary material for: Regulation of the Flavonoid Biosynthesis Pathway Genes in Purple and Black Grains of Hordeum vulgare
Source: PLoS One. 2016 Oct 5;11(10):e0163782. doi: 10.1371/journal.pone.0163782 (PMC5051897; doi:10.1371/journal.pone.0163782)
Supplement: S4 Fig — The residues differing in Bowman and PLP are marked by pink color. (DOCX) [file pone.0163782.s004.docx]

**S4 Fig. Secondary structure of the ANT2 protein of Bowman and PLP predicted by CFSSP Server.** The residues differing in Bowman and PLP are marked by pink color.

Name of the sequence is *Bowman* Sequence consists of 559 amino acids.

**Target Sequence:**

MALPIVRPSQ EEPPTGKQFS YQLAAAVRSI NWSYAIFWSI STSRPGVLTW KDGFYNGEIK TRKVTSSADL TADQLVLQRS EQLRELYQSL LSGQCDHRGR RPAAALSPED LGDAEWYYAV CMSYAFRPGQ GLPGRSFASN EPVWLCNAQC ADTKTFQRSL LAKTTSIQTV ACIPLMGGVL ELGTTDTVLE DRDMVNRIST SFWDLKIPTS SKPKEPSSPS ADDAGEADIV FQDLDHNTMA AMIPGELELG EVECLSDDNL ERITKEINGF YGLCDELDVG ALDENWIIGG SFEVMSSPEA PPAPAATGGI TDGIVTLSAA ASSLSSCFTA WKRSWDSAED MAAPVAGQSQ KLLKKALAGG AWAINGGGGG GTARAQESSN TKNHVISERR RREKLNEMFL ILKSLVPSIH KVDKASILAE TIAYLRELEQ RVEELESNRA PSRPAGAAVR RHHDAAAKKM LAGSKRKASE LGGDDGPNSV VNVTVMEKEV LLEVQCRWKE LLMTQVFDAI KSLRLDVLSV RASTPDGLLA LKIRAQFAGP GAVEPGMIIG ALQTATRGR


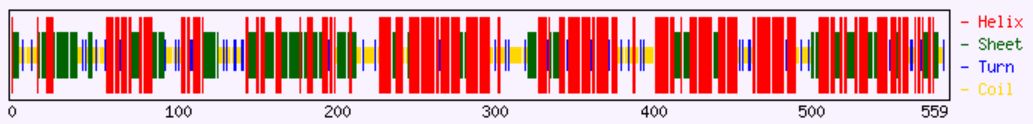


**Secondary Structure:**

* * * * * *

Query 1 MALPIVRPSQEEPPTGKQFSYQLAAAVRSINWSYAIFWSISTSRPGVLTWKDGFYNGEIKTRKVTSSADL 70

Helix 1 HH HHHHHHHHHHHHHHHHHHHHHHHH HH HHHHHHHHHHHHH 70

Sheet 1 EEEEE EEEEEEEEEEEEEEEEEEEEEEE EEE E 70

Turns 1 T T T TT T T T 70

Struc 1 HEEEECCTCCCCTCCCHEEEEHHHHHEEEEEEEEEEEEEECCCTTCEEECCTCCCCTHHHHHHHHTHHHH 70

* * * * * *

Query 71 TADQLVLQRSEQLRELYQSLLSGQCDHRGRRPAAALSPEDLGDAEWYYAVCMSYAFRPGQGLPGRSFASN 140

Helix 71 HHHHHHHHHHHHHHHHHHHH HHHHHHHHHHHHHHHHHH HH 140

Sheet 71 EEEEEEEEEEEEEEEEEEEEEEE EEEEEEEEE 140

Turns 71 T T T T T T TT T T T TT TT 140

Struc 71 HHEEEEEHHHHHHHHEEEEEEETCCCCCTCTCHHHHHTTHHHHHHEEEEEEEEECCCTCTCCCTTCCCTT 140

* * * * * *

Query 141 EPVWLCNAQCADTKTFQRSLLAKTTSIQTVACIPLMGGVLELGTTDTVLEDRDMVNRISTSFWDLKIPTS 210

Helix 141 HHHHHHHHHHHHHHHHHHHHHHHHHHHHHHHHHHHHHHHHHHHHHHHHHHHHHH HHHHHH 210

Sheet 141 EEEEEEEEEEEEEEEEEEEEEEEEEEEEEEEEEEEEEEEEEEEEEE EEEEEEEEEEEE 210

Turns 141 T T TT T 210

Struc 141 HHEEEEEHHHHHEEEEEEHHHHEEEEEEEEEEEHEEEHHHHEEEEEHHHHTHHHCEEEEEEEHHEEECCC 210

* * * * * *

Query 211 SKPKEPSSPSADDAGEADIVFQDLDHNTMAAMIPGELELGEVECLSDDNLERITKEINGFYGLCDELDVG 280

Helix 211 HHHHHHHHHHHHHHH HHHHHHHHHHHHHHHHHHHHHHHHHHHHHHH HHHHHHHH 280

Sheet 211 EEEEE EEEE EEEE 280

Turns 211 T T T TT T 280

Struc 211 TCCCCCCCTCHHHHHHHHEEHHHHHCCEHHHHHHHHHHHHHHHHHHTHHHHHHHHHHHHEEEHHHHHHHH 280

* * * * * *

Query 281 ALDENWIIGGSFEVMSSPEAPPAPAATGGITDGIVTLSAAASSLSSCFTAWKRSWDSAEDMAAPVAGQSQ 350

Helix 281 HHHHHHH HH HHHHHHHHHHHHHHHHHHHHHHHHHHHHHHHHHHHHH 350

Sheet 281 EEEEEEE EEEEEEE EEE 350

Turns 281 T T TT T T T T T T T T 350

Struc 281 HHHHHHHCCTCHHCCCTTCCCCCCCCCTCEEEEEEHHHHHHTHEEEEEHHHHTHHHHHHHHHHHHHHTHH 350

* * * * * *

Query 351 KLLKKALAGGAWAINGGGGGGTARAQESSNTKNHVISERRRREKLNEMFLILKSLVPSIHKVDKASILAE 420

Helix 351 HHHHHHHHHHHHH HH HHHHHHHHHHHHHHHHHHHHHHHHHHHHHHHHHHH 420

Sheet 351 EE EEEEEEEEEEEE EEEE 420

Turns 351 T TT T T T T T T 420

Struc 351 HHHHHHHHTHHHHCCTCCCTCCHHCCTCTCCCCCCHHHHHHHHHHHHEEEEHEEEEHHHHHHHHHHHHHH 420

* * * * * *

Query 421 TIAYLRELEQRVEELESNRAPSRPAGAAVRRHHDAAAKKMLAGSKRKASELGGDDGPNSVVNVTVMEKEV 490

Helix 421 HHHHHHHHHHHHHHH HHHHHHHHHHHHHHHHHHHHHHHHHH HHHHHHHHHHH 490

Sheet 421 EEEE EEEEEE E 490

Turns 421 T TT TT T T T T T 490

Struc 421 EEEHHHHHHHHHHHHCTTCCCTTCHHHHHHHHHHHHHHHHHHHTHHHHHHCCTCCCCTCEEEEHHHHHHH 490

* * * * * *

Query 491 LLEVQCRWKELLMTQVFDAIKSLRLDVLSVRASTPDGLLALKIRAQFAGPGAVEPGMIIGALQTATRGR 559

Helix 491 HHHHHHHHHHHHHHHHHHHHHHHHHHHHHHHHHHHHHHHHHHHHHHH HHHHHHHHHHHHH 559

Sheet 491 EEEEEEEEEEEEEEEEE EEEEEEEE EEEEEEEEE EEEEEEEEE 559

Turns 491 T T TT T T 559

Struc 491 HHHEEEHHHHEEEEEHHHHHHHHEEEEEHHHHHHHTHHHHEHHHHHHCTTCHHHTHEEEHHHEEECCCT 559

Total Residues: H: 403 E: 224 T: 71

Percent: H: 72.1 E: 40.1 T: 12.7

**S3 Fig.** *Cont.*

Name of the sequence is *PLP* Sequence consists of 559 amino acids.

**Target Sequence:**

MALPIVRPSQ EEPPTGKQFS YQLAAAVRSI NWSYAIFWSI STSRPGVLTW KDGFYNGEIK TRKVTSSADL TADQLLLQRS EQLRELYQSL LSGQCDHRGR RPAAALSPED LGDAEWYYAV CMSYAFRPGQ GLPGRSFASN EPVWLCNAQC ADTKTFQRSL LAKTTSIQTV ACIPLMGGVL ELGTTDTVLE DRDMVNRIST SFWDLKIPTS SKPKEPSSPS ADDAGEADIV FQDLDHNTMA AMIPGELELG EVECLSDDNL ERITKEIKRF YGLCDELDVG ALDENWIIGG SFEVMSSPEA PPAPAATGGI TDGIVTLSAA ASSLSSCFTA WKRSWDSAED MAAPVAGQSQ KLLKKALAGG VWAINGGGGG GTARAQESSN TKNHVISERR RREKLNEMFL ILKSLVPSIH KVDKASILAE TIAYLRELEQ RVEELESNRA PSRPAGAAVR RHHDAAAKKM LAGSKRKASE LGGDDGPNSV VNVTVTEKEV LLEVQCRWKE LLMTQVFDAF KSLRLDVLSV RASTPDGLLA LKIRAQFAGP GAVEPGMIIG ALQTATRGR


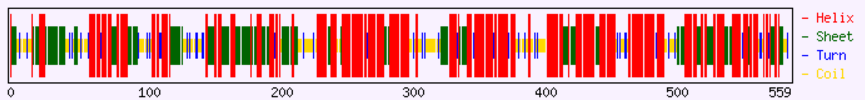


**Secondary Structure:**

* * * * * *

Query 1 MALPIVRPSQEEPPTGKQFSYQLAAAVRSINWSYAIFWSISTSRPGVLTWKDGFYNGEIKTRKVTSSADL 70

Helix 1 HH HHHHHHHHHHHHHHHHHHHHHHHH HH HHHHHHHHHHHHH 70

Sheet 1 EEEEE EEEEEEEEEEEEEEEEEEEEEEE EEE E 70

Turns 1 T T T TT T T T 70

Struc 1 HEEEECCTCCCCTCCCHEEEEHHHHHEEEEEEEEEEEEEECCCTTCEEECCTCCCCTHHHHHHHHTHHHH 70

* * * * * *

Query 71 TADQLLLQRSEQLRELYQSLLSGQCDHRGRRPAAALSPEDLGDAEWYYAVCMSYAFRPGQGLPGRSFASN 140

Helix 71 HHHHHHHHHHHHHHHHHHHH HHHHHHHHHHHHHHHHHH HH 140

Sheet 71 EEEEEEEEEEEEEEEEEEEEEEE EEEEEEEEE 140

Turns 71 T T T T T T TT T T T TT TT 140

Struc 71 HHHEEEEHHHHHHHHEEEEEEETCCCCCTCTCHHHHHTTHHHHHHEEEEEEEEECCCTCTCCCTTCCCTT 140

* * * * * *

Query 141 EPVWLCNAQCADTKTFQRSLLAKTTSIQTVACIPLMGGVLELGTTDTVLEDRDMVNRISTSFWDLKIPTS 210

Helix 141 HHHHHHHHHHHHHHHHHHHHHHHHHHHHHHHHHHHHHHHHHHHHHHHHHHHHHH HHHHHH 210

Sheet 141 EEEEEEEEEEEEEEEEEEEEEEEEEEEEEEEEEEEEEEEEEEEEEE EEEEEEEEEEEE 210

Turns 141 T T TT T 210

Struc 141 HHEEEEEHHHHHEEEEEEHHHHEEEEEEEEEEEHEEEHHHHEEEEEHHHHTHHHCEEEEEEEHHEEECCC 210

* * * * * *

Query 211 SKPKEPSSPSADDAGEADIVFQDLDHNTMAAMIPGELELGEVECLSDDNLERITKEIKRFYGLCDELDVG 280

Helix 211 HHHHHHHHHHHHHHH HHHHHHHHHHHHHHHHHHHHHHHHHHHHHHHHHHHHHHHHHH 280

Sheet 211 EEEEE EEEE EEEE 280

Turns 211 T T T TT T 280

Struc 211 TCCCCCCCTCHHHHHHHHEEHHHHHCCEHHHHHHHHHHHHHHHHHHTHHHHHHHHHHHHEEEHHHHHHHH 280

* * * * * *

Query 281 ALDENWIIGGSFEVMSSPEAPPAPAATGGITDGIVTLSAAASSLSSCFTAWKRSWDSAEDMAAPVAGQSQ 350

Helix 281 HHHHHHH HH HHHHHHHHHHHHHHHHHHHHHHHHHHHHHHHHHHHHH 350

Sheet 281 EEEEEEE EEEEEEE EEE 350

Turns 281 T T TT T T T T T T T T 350

Struc 281 HHHHHHHCCTCHHCCCTTCCCCCCCCCTCEEEEEEHHHHHHTHEEEEEHHHHTHHHHHHHHHHHHHHTHH 350

* * * * * *

Query 351 KLLKKALAGGVWAINGGGGGGTARAQESSNTKNHVISERRRREKLNEMFLILKSLVPSIHKVDKASILAE 420

Helix 351 HHHHHHHHHHHHH HH HHHHHHHHHHHHHHHHHHHHHHHHHHHHHHHHHHH 420

Sheet 351 EE EEEEEEEEEEEE EEEE 420

Turns 351 T T T T T T T T 420

Struc 351 HHHHHHHHTHHHHCCTCCCTCCHHCCTCTCCCCCCHHHHHHHHHHHHEEEEHEEEEHHHHHHHHHHHHHH 420

* * * * * *

Query 421 TIAYLRELEQRVEELESNRAPSRPAGAAVRRHHDAAAKKMLAGSKRKASELGGDDGPNSVVNVTVTEKEV 490

Helix 421 HHHHHHHHHHHHHHH HHHHHHHHHHHHHHHHHHHHHHHHHH HHHHHHHHHHH 490

Sheet 421 EEEE EEEEEEEEEEE 490

Turns 421 T TT TT T T T T T 490

Struc 421 EEEHHHHHHHHHHHHCTTCCCTTCHHHHHHHHHHHHHHHHHHHTHHHHHHCCTCCCCTCEEEEEHHHHHH 490

* * * * * *

Query 491 LLEVQCRWKELLMTQVFDAFKSLRLDVLSVRASTPDGLLALKIRAQFAGPGAVEPGMIIGALQTATRGR 559

Helix 491 HHHHHHHHHHHHHHHHHHHHHHHHHHHHHHHHHHHHHHHHHHHHHHH HHHHHHHHHHHHH 559

Sheet 491 EEEEEEEEEEEEEEEEE EEEEEEEE EEEEEEEEE EEEEEEEEE 559

Turns 491 T T TT T T 559

Struc 491 HHHEEEHHHHEEEEEHHHHHHHHEEEEEHHHHHHHTHHHHEHHHHHHCTTCHHHTHEEEHHHEEECCCT 559

Total Residues: H: 406 E: 228 T: 70

Percent: H: 72.6 E: 40.8 T: 12.5
