# Supplementary material for: Regulation of the Flavonoid Biosynthesis Pathway Genes in Purple and Black Grains of Hordeum vulgare
Source: PLoS One. 2016 Oct 5;11(10):e0163782. doi: 10.1371/journal.pone.0163782 (PMC5051897; doi:10.1371/journal.pone.0163782)
Supplement: S5 Fig — Exonic sequences are marked by green color. (DOCX) [file pone.0163782.s005.docx]

**S5 Fig. Alignment of the nucleotide sequences of the 5’ regulatory region of the *Ant2* gene of Bowman and PLP line.** Exonic sequences are marked by green color.

**BOWMAN GCCGTGTGTTTCCTTAGTTGTCGTTGACGTTGATTATTCTGTGCCTGTTACCCCATCACCTCAGCGTCAAGCAGAGATCGGTGAGGATTGCGTACATCTCACTCCATTTTTATCATAGTTTTAAA**

**PLP GCCGTGTGTTTCCTTAGTTGTCGTCGACGTCGATTATTCTGTGCCTGTTACCCCGTCACCTCAGCGTCAAGCAGAGATCGGTGAGGATTGCGTGCATCTCACTCCATTTTTA**

**BOWMAN TAGCCGGCTATAGTCCCGCTATAACCTTTTCAATAGGGTGCCGCTAAATGGTATCACGTACAAATATGCCGCTATAGCCCGATATAGCCCGCTATAGCTCCGCTATAGCTGATTTTCAGGCATGC**

**PLP**

**BOWMAN CGCTATTTGTCATAGCCCGCTATTTAAAACATTGATTTTAACTAAGCACTGAGAAGACCAAGCAGGCATGCACACCAAACTAATCAGAAAGTGATGGGCCTAATATGAAACTAATCAGAAGTACC**

**PLP CTAAGCAGTGAGAAGACCAAGCAGGCATGCACACCAAACTAATCAGAAAGTGATGGGCCTAATATGAAACTAATCAGAAGTACC**

TATA box

**BOWMAN ATGTTGGAGCACTACAGTAGTGTCCGGACAGTACTTTGCTTTTGCGTCGCAGTCTCGCTTGTTGTTGGCTCGTTGCTCGGAGCCTCGGACAGATCTATATAACATGGCTTGGATTCTAGATACAT**

**PLP ATGTTGGAGCACTACAGTAGTGTCCGGACAGTACTTTGCTTTTGCGTCGCAGTCTCGCTTGTTG GCTCGTTGCTCGGAGCCTCGGACAGATCTATATAACATGCCCTGGATTCTAGATACAT**

**BOWMAN TGCACATATAGAAGCTCCTTCTTCCTCTCCGGACGACAGGTTGGACTCCGGGCTTCTTGGTCTCCATAGCTCAAAGTAAACCTTTAACCTCCTCTTAGATGGAAAATTAATCCTCCACTCATGTC**

**PLP TGCACATATAGAAGCTCCTTCTTCCTCTCCGGACGACAGGTTGGACTCCGGGCTTCTTGGTCTCCATAGCTCAAAGTAAACCTTTAACCTCCTCTTAGATGGAAAATTAATCCTCCACTCATGTC**

**BOWMAN TAGCTTAGTTTATCTCTTACTTTCTTGGTTTGTTCCAGCTTTCGATCTAGTATATACAGTATGTTCTTTGTAATCTTCGTTGAGAAATCTTCCCAGGAGTTTCTTAATTAGCACCAGTGCAACAG**

**PLP TAGCTTAGTTTATCTCTTACTTTCTTGGTTTGTTCCAGCTTTCGATCGAGTATATACAGTATGTTCTTTGTAATCTTCGTTGAGAAATCTTCCCAGGAGTTTCTTAATTAGCACCAGTGCAACAG**

**BOWMAN AGTGGAGGGATTTCGATCAGCTAGCATGAACTAAATTATCCAAGAAGATGCATCATGCTCACTATAAACACAAGAGATGGAACCATATATAGCTTCATTGATTTGTTTATTTTTGCCTCCTTGCT**

**PLP AGTGGAGGGATTTCGATCAGCTAGCATGAACTAAATTATCCAAGAAGATGCATCATGCTCACTATAAACACAAGAGATGGAACCATATATAGCTTCATTGATTTGTTTATTTTTGCCTCCTTGCT**

exon 1

**BOWMAN TGCTCTAACTTCACCTCGTCTGCATCATGCATATGAAATGAAGGAAGGAAATAATATGGTAATGGCGCTACCA**

**PLP TGCTCTAACTTCACCTCGTCTGCATCATGCATATGAAATGAAGGAAGGAAATAATATGGTAATGGCGCTACCA**
