# Supplementary material for: Regulation of the Flavonoid Biosynthesis Pathway Genes in Purple and Black Grains of Hordeum vulgare
Source: PLoS One. 2016 Oct 5;11(10):e0163782. doi: 10.1371/journal.pone.0163782 (PMC5051897; doi:10.1371/journal.pone.0163782)
Supplement: S6 Fig — The overlay of chromatograms obtained by extracting the PDA data at 280 from extract 1 of all genotypes is shown. Chromatograms are depicted in black (Bowman), blue (PLP) and red (BLP). (DOCX) [file pone.0163782.s006.docx]

**S6 Fig. Phenylpropanoid profiles of Bawman, PLP and BLP seed extracts.** The overlay of chromatograms obtained by extracting the PDA data at 280 from extract 1 of all genotypes is shown. Chromatograms are depicted in black (Bowman), blue (PLP) and red (BLP).

- Bowman

- PLP

- BLP

[Geben Sie ein Zitat aus dem Dokument oder die Zusammenfassung eines interessanten Punkts ein. Sie können das Textfeld an einer beliebigen Stelle im Dokument positionieren. Verwenden Sie die Registerkarte 'Zeichentools', wenn Sie das Format des Textfelds 'Textzitat' ändern möchten.]
