# Supplementary material for: Regulation of the Flavonoid Biosynthesis Pathway Genes in Purple and Black Grains of Hordeum vulgare
Source: PLoS One. 2016 Oct 5;11(10):e0163782. doi: 10.1371/journal.pone.0163782 (PMC5051897; doi:10.1371/journal.pone.0163782)
Supplement: S1 Table — (DOCX) [file pone.0163782.s007.docx]

**S1 Table. Data on the structural and regulatory genes for flavonoid biosynthesis in barley identified up to date.**

| **№** | **Gene nаme** | **Cloning approach** | **Accession number** | **Genome localization** |
| --- | --- | --- | --- | --- |
| **1** | Chalcone synthase – *Chs* | 1full-lenght cDNA copy, 1 full-length gDNA copy, | Y09233 [6]; X58339 [8] | 1HS [9]; 1HS, 1HL, 6HS [10] |
| **2** | Chalcone-flavanone isomerase – *Chi* | 1 full-length gDNA copy | AF474923 [11] | 5HL [11] |
| **3** | Flavanone 3-hydroxylase – *F3h* | 1 full-length cDNA copy | X58138 [12] | 2HL [10, 13] |
| **4** | Flavonoid 3'-hydroxylase – *F3’h* | 1 full-length cDNA copy, gDNA copies from database | AK363912 [39]; Morex contig 1575828^a^, Barke contig 2826620^a^, Bowman contig 404^a^ (S1 File) | 1H^a^ |
| **5** | Dihydroflavanol reductase – *Dfr* | 1 full-length cDNA copy | S69616 [14] | 3HL [10] |
| **6** | Anthocyanidin synthase – *Ans* | 1 full-length gDNA copy from database | Bowman contigs 941389, 1630795, 1993730^a^ (S2 File) | 5HL^a^ |
| **7** | 3,4-*cis*-Leucoanthocyanidin reductase – *Lar* | 1 full-length cDNA copy | BN000696 [15] | – |
| **8** | UDP glucose:flavonol 3- -glucosyltransferase – *Ufgt* | 1 full-length gDNA copy | X15694 [16] | 7HS [16] |
| **9** | *Anthocyanin-less 1 – Ant1* | 1 full-length gDNA copy | KP265976-79 [18]; [17] | 7HS [17] |
| **10** | *Anthocyanin-less 2 – Ant2* | 1 full-length gDNA copy | HM370298–387 [19] | 2HL [19] |
| **11** | *Anthocyanin-less 28 – Ant28* | 1 full-length gDNA copy | AB645844 [20] | 3HL [20, 26] |

^a^ The contigs corresponding to the gene were identified in the BARLEX database in the current study
