# Supplementary material for: Regulation of the Flavonoid Biosynthesis Pathway Genes in Purple and Black Grains of Hordeum vulgare
Source: PLoS One. 2016 Oct 5;11(10):e0163782. doi: 10.1371/journal.pone.0163782 (PMC5051897; doi:10.1371/journal.pone.0163782)
Supplement: S2 Table — (DOCX) [file pone.0163782.s008.docx]

**S2 Table. Primer pairs amplifying *Ant2* gene 5’ regulatory region designed in the current study.**

| № | Forward primer (5’→3’) | Reverse primer (5’→3’) | Bowman, bp | PLP, bp |
| --- | --- | --- | --- | --- |
| 1 | GCCGTGTGTTTCCTTAGTT | CGAGCCAACAACAAGCGAGAC | 447 | 268 |
| 2 | GTACTTTGCTTTTGCGTC | TGGGAAGATTTCTCAACGAAG | 315 | 312 |
| 3 | TCGCAGTCTCGCTTGTTGT | TGGGAAGATTTCTCAACGAAG | 299 | 299 |
