# Supplementary material for: Regulation of the Flavonoid Biosynthesis Pathway Genes in Purple and Black Grains of Hordeum vulgare
Source: PLoS One. 2016 Oct 5;11(10):e0163782. doi: 10.1371/journal.pone.0163782 (PMC5051897; doi:10.1371/journal.pone.0163782)
Supplement: S3 Table — (DOCX) [file pone.0163782.s009.docx]

**S3 Table. Amino acid substitutions identified between ANT2 of Bowman and PLP and its classification based on the polarity of the side chain group.**

| **№** | **Residue** | **Bowman** | **PLP** |
| --- | --- | --- | --- |
| 1 | 76 | Val (V)  nonpolar | Leu (L)  nonpolar |
| 2 | 268 | Asn (N)  polar, no charged | Lys (K)  polar, positively charged at pH=7 |
| 3 | 269 | Gly (G)  polar, no charged | Arg (R)  polar, positively charged at pH=7 |
| 4 | 361 | Ala (A)  nonpolar | Val (V)  nonpolar |
| 5 | 486 | Met (M)  nonpolar | Thr (T)  polar, no charged |
| 6 | 510 | Ile (I)  nonpolar | Phe (F)  nonpolar |
