# Supplementary material for: Regulation of the Flavonoid Biosynthesis Pathway Genes in Purple and Black Grains of Hordeum vulgare
Source: PLoS One. 2016 Oct 5;11(10):e0163782. doi: 10.1371/journal.pone.0163782 (PMC5051897; doi:10.1371/journal.pone.0163782)
Supplement: S4 Table — (DOCX) [file pone.0163782.s010.docx]

**S4 Table. Putative *cis*-acting regulatory elements identified in promoter regions of Bowman (936 bp before ATG codon) and PLP (754 bp before ATG codon) *Ant2* genes using the PLACE database.** Similar *cis*-elements in Bowman and PLP are green, unique *cis*-elements are red, *cis*-elements placed in Bowman insertion are gray. TF – transcription factor.

| **Stimuli / transcriptional factors** | **Name of the *cis*-regulatory element** | **Description** | **Sequence** | **Bowman** | **PLP** | **References** |
| --- | --- | --- | --- | --- | --- | --- |
|  | TATA box | TATA box found in the 5'upstream region of sweet potato sporamin A gene and beta-phaseolin promoter; Sequence and spacing of TATA box elements are critical for accurate initiation | TATATAA | 471 (+) | 289 (+) | Grace et al., 2004 |
| **light** | SORLIP2 | One of "Sequences Over-Represented in Light-Induced Promoters (SORLIPs) in *Arabidopsis*; Computationally identified *phyA*-induced motifs | GGGCC | 346 (+) | 167 (+) | Hudson and Quail, 2003 |
|  | SORLIP5 | One of "Sequences Over-Represented in Light-Induced Promoters (SORLIPs) in *Arabidopsis*; Computationally identified *phyA*-induced motifs; Over-represented in both light-induced cotyledon-specific and root-specific genes | GAGTGAG | 98 (-) | 98 (-) | Hudson and Quail, 2003;  Jiao et al., 2005 |
|  | GT-1 site | Consensus GT-1 binding site in many light-regulated genes, e.g., *RBSC* from many species, *PHYA* from oat and rice, spinach *RCA* and *PETA*, and bean *CHS15*; GT-1 can stabilize the TFIIA-TBP-DNA (TATA box) complex; The activation mechanism of GT-1 may be achieved through direct interaction between TFIIA and GT-1 | GRWAAW;  R=A/G;  W=A/T | 109 (-);  237 (-) |  | Terzaghi and Cashmore, 1995;  Villain et al., 1996;  Gourrierec et al., 1999;  Buchel et al., 1999;  Zhou, 1999 |
|  | I Box | Conserved sequence upstream of light-regulated genes; Conserved sequence upstream of light-regulated genes of both monocots and dicots | GATAA | 110 (-) |  | Terzaghi and Cashmore,1995 |
|  | T Box | "T box" found in the *Arabidopsis thaliana* *GAPB* gene promoter, encoding the B subunit of chloroplast glyceraldehyde-3-phosphate dehydrogenase; Located between -94 and -89 (T1) and also between -84 and -79 (T2); Mutations in the "T box" resulted in reductions of light-activated gene transcription | ACTTTG | 408 (+) | 229 (+) | Chan et al., 2001 |
|  | CAANNNNATC motif | Region necessary for circadian expression of tomato light-harvesting complex protein genes (*Lhc*); Motif CAANNNNATC is conserved in 5′-upstream regions of clock controlled *Lhc* genes and overlaps with a sequence relevant in phytochrome mediated gene expression | CAANNNNATC | 326 (+) | 147 (+) | Piechulla et al., 1998 |
| **dehydration** | ABRE-like sequence | ABRE-like sequence (from -199 to -195) required for etiolation-induced expression of *erd1* (early responsive to dehydration) in *Arabidopsis* | ACGTG | 180 (-) |  | Simpson et al., 2003;  Nakashima et al., 2006 |
|  | ACGT sequence | ACGT sequence (from -155 to -152) required for etiolation-induced expression of erd1 (early responsive to dehydration)in *Arabidopsis* | ACGT | 27 (-/+);  181 (-/+) | 27 (-/+) | Simpson et al., 2003 |
|  | C-repeat | Binding site of barley C-repeat binding factors CBF1 and CBF2; CBFs are also known as dehydration-responsive element (DRE) binding proteins (DREBs) | RYCGAC;  R=A/G;  Y=C/T |  | 23 (-/+);  29 (-) | Xue 2002;  Svensson et al., 2006 |
| **WRKY (pathogen- and SA-induced DNA binding proteins)** | W-box | "W-box" found in promoter of *Arabidopsis thaliana* *NPR1* gene; Located between +70 and +79 in tandem; They were recognized specifically by salicylic acid (SA)-induced WRKY DNA binding proteins | TTGAC | 24 (+);  66 (-) | 66 (-) | Yu et al., 2001;  Chen et al., 2002;  Chen and Chen, 2002;  Maleck et al., 2000;  Xu et al., 2006 |
|  | core of TGAC-containing W-box | "A core of TGAC-containing W-box" of, e.g., *Amy32b* promoter; Binding site of rice WRKY71, a transcriptional repressor of the gibberellin signaling pathway; Parsley WRKY proteins bind specifically to TGAC-containing W box elements ( TTGAC, TGACT) within the Pathogenesis-Related Class10 (PR-10) genes | TGAC | 25 (+);  259 (-) | 55 (-);  66 (-) | Zhang et al., 2004;  Xie et al., 2005;  Eulgem et al., 1999;  Eulgem et al., 2000 |
| **cytokinin** | ARR1-binding element | "ARR1-binding element" found in *Arabidopsis*; ARR1 is a cytokinin-regulated TF; AGATT is found in the promoter of rice non-symbiotic haemoglobin-2 (*NSHB*) gene | NGATT;  N=G/A/C/T | 31 (+);  85 (+);  235 (+);  332 (-);  363 (-) | 31 (+);  85 (+);  153 (-);  184 (-) | Sakai et al., 2000;  Ross et al., 2004 |
|  | CPBE - Cytokinin-enhanced Protein Binding Element | The sequence critical for Cytokinin-enhanced Protein Binding *in vitro*, found in the promoter of the cucumber NADPH-protochlorophyllide reductase gene; Function effectively when interacting with other cytokinin-related elements | TATTAG | 350 (-) | 171 (-) | Fusada et al., 2005 |
| **bZIP** | DPBF binding site | Binding sequence for bZIP TFs, DPBF-1 and -2 (Dc3 Promoter-Binding Factor-1 and 2), found in promoter of the carrot *Dc3* gene which is belonged to late embryogenesis-abundant class genes; *Dc3* expression can be induced by ABA; In *Arabidopsis*, orthologous gene *ABI5*encoding for a bZIP TF regulates a subset of late embryogenesis-abundant genes | ACACNNG | 3 (-) | 3 (-) | Kim et al., 1997;  Finkelstein and Lynch, 2000;  Lopez-Molina and Chua, 2000 |
|  | As-1 - activating sequence-1 | "ASF-1 binding site" in CaMV 35S promoter; ASF-1 binds to two TGACG motifs; TGACG motifs are found in many promoters and are involved in transcriptional activation of several genes by auxin and/or salicylic acid; May be relevant to light regulation; Binding site of tobacco TGA1a (a member of the bZip family of TFs; Abiotic and biotic stress differentially stimulate "as-1 element" activity | TGACG | 25 (+);  65 (-) | 54 (-);  65 (-); | Despres et al., 2003;  Terzaghi and Cashmore, 1995;  Benfey and Chua, 1990;  Katagiri et al, 1989;  Xiang et al., 1997;  Klinedinst et al., 2000;  Redman et al., 2002 |
|  | C-box | "C-box" according to the nomenclature of ACGT elements by Foster et al. (1994); One of ACGT elements; Factors groups 2 and 3 have affinity for C-box | GACGTC |  | 26 (-/+) | Foster et al., 1994;  Izawa et al., 1994;  Izawa et al., 1993 |
| **MYB** | MRE (MYB recognition element) | Binding site for all animal MYB and at least two plant MYB proteins ATMYB1 and ATMYB2, both isolated from *Arabidopsis*; ATMYB2 is involved in regulation of genes that are responsive to water stress in *Arabidopsi*s; A petunia MYB protein is involved in regulation of flavonoid biosynthesis | CNGTTR;  N=G/A/C/T;  R=A/G | 45 (+) | 45 (+) | Luscher and Eisema, 1990;  Urao et al., 1993;  Solano et al., 1995 |
|  | MYB binding site | Plant MYB binding site; Consensus sequence related to box P in promoters of phenylpropanoid biosynthetic genes such as *Pal*, *Chs*, *Chi*, *Dfr*, *Cl*, *Bz1*; The AmMYB308 and AmMYB330 TFs from *Antirrhinum majus* regulate phenylpropanoid and lignin biosynthesis in transgenic tobacco; | MACCWAMC  M=A/C;  W=A/T | 323 (+) | 144 (+) | Sablowski et al., 1994;  Tamagnone et al., 1998 |
| **BELL** | TGTCA motif | Binding site of OsBIHD1, a rice BELL homeodomain TF; | TGTCA | 258 (+) |  | Luo et al., 2005 |
| **DOF** | recognition core of Dof proteins | Core site required for binding of unique to plants Dof (DNA binding with one finger) proteins, having diverse roles in gene expression when associated with plant-specific phenomena including light, phytohormone and defense responses, seed development and germination | AAAG | 151 (-);  338 (+);  409 (-);  414 (-) | 159 (+);  230 (-)  235 (-) | Yanagisawa and Schmidt, 1999;  Yanagisawa, 2000 |

**References**

Benfey PN, Chua NH. The cauliflower mosaic virus 35S promoter: combinatorial regulation of transcription in plants Science 250:959-966 (1990)

Buchel AS, Brederode FT, Bol JF, Linthorst HJM Mutation of GT-1 binding sites in the Pr-1A promoter influences the level of inducible gene expression in vivo Plant Mol Biol 40:387-396 (1999)

Chan CS, Guo L, Shih MC. Promoter analysis of the nuclear gene encoding the chloroplast glyceraldehyde-3-phosphate dehydrogenase B subunit of *Arabidopsis thaliana*. Plant Mol Biol 46: 131-141 (2001)

Chen W, Provart NJ, Glazebrook J, Katagiri F, Chang HS, Eulgem T, Mauch F, Luan S, Zou G, Whitham SA, Budworth PR, Tao Y, Xie Expression profile matrix of Arabidopsis transcription factor genes suggests their putative functions in response to environmental stresses. Plant Cell 14: 559-574 (2002)

Chen C, Chen Z. Potentiation of developmentally regulated plant defense response by AtWRKY18, a pathogen-induced Arabidopsis transcription factor. Plant Physiol. 129:706-716 (2002)

Despres C, Chubak C, Rochon A, Clark R, Bethune T, Desveaux D, Fobert PR. The Arabidopsis NPR1 disease resistance protein is a novel cofactor that confers redox regulation of DNA binding activity to the basic domain/leucine zipper transcription factor TGA1. Plant Cell 15: 2181-2191 (2003)

Eulgem T, Rushton PJ, Robatzek S, Somssich IE. The WRKY superfamily of plant transcription factors. Trends Plant Sci. 5:199-206. (2000)

Eulgem T, Rushton PJ, Schmelzer E, Hahlbrock K, Somssich IE. Early nuclear events in plant defence signalling: rapid gene activation by WRKY transcription factors. EMBO J. 18:4689-4699 (1999)

Finkelstein RR, Lynch TJ. The Arabidopsis abscisic acid response gene ABI5 encodes a basic leucine zipper transcription factor. Plant Cell 12: 599-609 (2000)

Foster R, Izawa T, Chua N-H. Plant bZIP Proteins gather at ACGT elements. FASEB J 8:192-200 (1994)

Fusada N, Masuda T, Kuroda H, Shimada H, Ohta H, Takamiya K. Identification of a Novel Cis-Element Exhibiting Cytokinin-Dependent Protein Binding in Vitro in the 5'-region of NADPH-Protochlorophyllide Oxidoreductase Gene in Cucumber. Plant Mol Biol. 59: 631-645 (2005).

Grace ML, Chandrasekharan MB, Hall TC, Crowe AJ. Sequence and spacing of TATA box elements are critical for accurate initiation from the beta-phaseolin promoter.J Biol Chem. 279:8102-8110 (2004).

Hudson ME, Quail PH. Identification of promoter motifs involved in the network of phytochrome A-regulated gene expression by combined analysis of genomic sequence and microarray data. Plant Physiol. 133: 1605-1616 (2003)

Izawa T, Foster R, Nakajima M, Shimamoto K, Chua N-H. The rice bZIP transcriptional activator RITA-1 is highly expressed during seed development. Plant Cell 6:1277-1287 (1994)

Izawa T, Foster R, Chua N-H. Plant bZIP protein DNA binding specificity. J Mol Biol 230:1131-1144 (1993)

Jiao Y, Ma L, Strickland E, Deng XW. Conservation and Divergence of Light-Regulated Genome Expression Patterns during Seedling Development in Rice and Arabidopsis. Plant Cell. 17: 3239-3256 (2005)

Katagiri F, Lam E, Chua NH. Two tobacco DNA-binding proteins with homology to the nuclear factor CREB Nature 31: 727-730 (1989)

Kim SY, Chung HJ, Thomas TL. Isolation of a novel class of bZIP transcription factors that interact with ABA-responsive and embryo-specification elements in the Dc3 promoter using a modified yeast one-hybrid system. Plant J 11: 1237-1251 (1997)

Klinedinst S, Pascuzzi P, Redman J, Desai M, Arias J. A xenobiotic-stress-activated transcription factor and its cognate target genes are preferentially expressed in root tip meristems. Plant Mol Biol 42: 679-688 (2000)

Le Gourrierec J, Li YF, Zhou DX Transcriptional activation by Arabidopsis GT-1 may be through interaction with TFIIA-TBP-TATA complex. Plant J 18:663-668 (1999)

Lopez-Molina L, Chua NH. A null mutation in a bZIP factor confers ABA-insensitivity in Arabidopsis thaliana. Plant Cell Physiol 41: 541-547 (2000)

Luo H, Song F, Goodman RM, Zheng Z. Up-regulation of OsBIHD1, a rice gene encoding BELL homeodomain transcriptional factor, in disease resistance responses. Plant Biol (Stuttg). 7: 459-468 (2005).

Luscher B, Eiseman RN. New light on Myc and Myb. Part II. Myb. Genes Dev. 4:2235-2241 (1990)

Maleck K, Levine A, Eulgem T, Morgan A, Schmid J, Lawton KA, Dangl JL, Dietrich RA. The transcriptome of *Arabidopsis thaliana* during systemic acquired resistance. Nat Genet. 26:403-410 (2000)

Piechulla B, Merforth N, Rudolph B. Identification of tomato Lhc promoter regions necessary for circadian expression. Plant Mol Biol 38:655-662 (1998)

Redman J, Whitcraft J, Johnson C, Arias J. Abiotic and biotic stress differentially stimulate as-1 element activity in Arabidopsis. Plant Cell Rep. 21: 180-185 (2002)

Ross EJ, Stone JM, Elowsky CG, Arredondo-Peter R, Klucas RV, Sarath G. Activation of the Oryza sativa non-symbiotic haemoglobin-2 promoter by the cytokinin-regulated transcription factor, ARR1. J Exp Bot. 55: 1721-1731 (2004).

Sablowski RWM, Moyano E, Culianez-Macia FA, Schuch W, Martin C, Bevan M A flower-specific Myb protein activates transcription of phenylpropanoid biosynthetic genes. EMBO J 13:128-137 (1994)

Sakai H, Aoyama T, Oka A. Arabidopsis ARR1 and ARR2 response regulators operate as transcriptional activators. Plant J. 24: 703-711 (2000).

Simpson SD, Nakashima K, Narusaka Y, Seki M, Shinozaki K, Yamaguchi-Shinozaki K. Two different novel cis-acting elements of erd1, a clpA homologous Arabidopsis gene function in induction by dehydration stress and dark-induced senescence. Plant J. 33: 259-270 (2003)

Solano R, Nieto C, Avila J, Canas L, Diaz I, Paz-Ares J. Dual DNA binding specificity of a petal epidermis-specific MYB transcription factor (MYB.Ph3) from *Petunia hybrid*. EMBO J 14:1773-1784 (1995)

Svensson JT, Crosatti C, Campoli C, Bassi R, Stanca AM, Close TJ, Cattivelli L. Transcriptome analysis of cold acclimation in barley albina and xantha mutants. Plant Physiol. 141:257-270. (2006)

Tamagnone L, Merida A, Parr A, Mackay S, Culianez-Macia FA, Roberts K, Martin C. The AmMYB308 and AmMYB330 transcription factors from antirrhinum regulate phenylpropanoid and lignin biosynthesis in transgenic tobacco. Plant Cell 10: 135-154 (1998)

Terzaghi WB, Cashmore AR Light-regulated transcription. Annu Rev Plant Physiol Plant Mol Biol 46:445-474 (1995)

Urao T, Yamaguchi-Shinozaki K, Urao S, Shinozaki K. An Arabidopsis myb homolog is induced by dehydration stress and its gene product binds to the conserved MYB recognition sequence Plant Cell 5:1529-1539 (1993)

Villain P, Mache R, Zhou DX The mechanism of GT element-mediated cell type-specific transcriptional control. J Biol Chem 271:32593-32598 (1996)

Xiang C, Miao Z, Lam E. DNA-binding properties, genomic organization and expression pattern of TGA6,a new member of the TGA family of bZIP transcription factors in Arabidopsis thaliana. Plant Mol Biol 34: 403-415 (1997)

Xie Z, Zhang ZL, Zou X, Huang J, Ruas P, Thompson D, Shen QJ. Annotations and functional analyses of the rice WRKY gene superfamily reveal positive and negative regulators of abscisic acid signaling in aleurone cells. Plant Physiol. 137:176-189 (2005)

Xu X, Chen C, Fan B, Chen Z. Physical and Functional Interactions between Pathogen-Induced Arabidopsis WRKY18, WRKY40, and WRKY60 Transcription Factors. Plant Cell 18:1310-1326 (2006)

Xue GP. Characterisation of the DNA-binding profile of barley HvCBF1 using an enzymatic method for rapid, quantitative and high-throughput analysis of the DNA-binding activity. Nucleic Acids Res. 30: e77 (2002)

Yanagisawa S, Schmidt RJ. Diversity and similarity among recognition sequences of Dof transcription factors. Plant J 17:209-214 (1999)

Yanagisawa S. Dof1 and Dof2 transcription factors are associated with expression of multiple genes involved in carbon metabolism in Maize. Plant J 21:281-288 (2000)

Yu D, Chen C, Chen Z. Evidence for an important role of WRKY DNA binding proteins in the regulation of NPR1 gene expression. Plant Cell 13: 1527-1540 (2001)

Zhang ZL, Xie Z, Zou X, Casaretto J, Ho TH, Shen QJ. A rice WRKY gene encodes a transcriptional repressor of the gibberellin signaling pathway in aleurone cells. Plant Physiol. 134:1500-1513(2004)

Zhou DX Regulatory mechanism of plant gene transcription by GT-elements and GT-factors. Trends in Plant Science 4:210-214 (1999)
