# Supplementary material for: Regulation of the Flavonoid Biosynthesis Pathway Genes in Purple and Black Grains of Hordeum vulgare
Source: PLoS One. 2016 Oct 5;11(10):e0163782. doi: 10.1371/journal.pone.0163782 (PMC5051897; doi:10.1371/journal.pone.0163782)
Supplement: S5 Table — Peaks areas of PDA data at 515 nm were integrated. The sum of all peak areas was taken for calculating total anthocyanin contents. A calibration curve using authentic cyanidin 3-O-glucoside was used. (DOCX) [file pone.0163782.s011.docx]

**S5 Table. Quantification of total anthocyanins in grains of Bowman, PLP and BLP.** Peaks areas of PDA data at 515 nm were integrated. The sum of all peak areas was taken for calculating total anthocyanin contents. A calibration curve using authentic cyanidin 3-*O*-glucoside was used.

| Genotype | Anthocyanin content  (ng/mg dry weight) | |
| --- | --- | --- |
|  | Extraction 1 | Extraction 2 |
| Bowman | 1.05 | 0.94 |
| PLP | 9.23 | 9.44 |
| BLP | 1.06 | 1.08 |
