# Supplementary material for: Regulation of the Flavonoid Biosynthesis Pathway Genes in Purple and Black Grains of Hordeum vulgare
Source: PLoS One. 2016 Oct 5;11(10):e0163782. doi: 10.1371/journal.pone.0163782 (PMC5051897; doi:10.1371/journal.pone.0163782)
Supplement: S1 File — (DOCX) [file pone.0163782.s012.docx]

**S1 File. The nucleotide sequences of Morex, Barke, and Bowman contigs corresponding to the *F3’h* gene found in BARLEX database.**

**>morex_contig_1575828 CAJW011575828 carma=1H**

TAGATTGACCGCGGTGGGTGGGGGCGGACGCCGAGGGGGCGCGCGCGTCCGCTTCGCGTTCGCGCCGACGCATTTCAGTCCAATTTAGACCGAAAATGGATCGACGCGGACGCGTTGTGGTAATGGATCGGCGCGTTGGGTCATCTTTTTTGTCCGTGCCGACCCAAACGAAGAGCGGCCGACGAAATATATTGCCCCGTTGGAGTTGCTCTTAGTGATGCAGACGCAGAGTTCATTGGGCCCCTTGAGGAGGAGGAGGAGGAGGAGGAGGAGGAGGAGGTTGGCAAGTCAATTTTGGAAAGCTCAGTTCTTGTCTTTGCTAGTCGCTTTTTTCCCATGTATATGTGGAGTGGAGTGGAGTAGACAGTGTAACAGCTACTGTACGTACGCCACTAGTCAGCCAGCCTCTCTGCAGCTGCACTGTTGAGCTACAGGAATAGGAGTAGGAGTAGGTAGGAGGAGCGGCCGCCCTTCCTTCCTTCCTGTTAGCTGGTGTGGTCGGCTACGTGCCATTCGCGCCATCAAACACAAGGCCCAATAATCGTTCGATGGAGCGACGGATGAGGAGGATCTGGCGATGCTGTTCGATGTAGTGTCAAGGAGGAAGAGAGGGTCGTTGGCTACCGTCAGCAAGGCAATGGAAGGAGAAGATAAATTGTAGCCTTTTAGGCAGCGCAAGCATGTGGCAAAGATGACTACCATTAGGGTTAGGACCTAGCTAGTATCATTTTGGTCTTCACCCTCCATCTAGCAGACACCAACACAAGTGACAAAAATCAGATTGATTAACCTTCATTCGAGAAGAAAAAGATAAAATTCCATTATAGCGGACAAAAAAATTGATGGATCCGGCCCATGTTTTCCCCCTCAACTTGGGAGTTTTCCATGTAAAAATACTGGCGTTTCAATGCGAGAGTTTTGAAGAACGTGTCTTCTATGCCAAATGGAATAATTAGTTACATTAAGACAAGAATTTTAAGACGGAAAGAGTAATAAGTAGTACTGATTATTCTCCATGAGCTGACGAGGAATTTGGTTGCGTTCTCACATAAGAATGTGCACTGCTAACCCTCAAAGATTCTGTCACCCCAAGTGGGTTATATAGCCAACAAAACGCCAACACATACAATGTGTACATTATAATTGTTAAGCAACAAAACCAGAAATATGAGGAGGGAAGTGACGTTGACTGTACCTCTCCAATCGTCCTGCCTTCCACTCCCCCTCAAAAAAACAAAACGTTCTTCCCTTTTTTACTAGAGCATATACTCCCTTCATAAACTATTAATATGAAAAAAAATAGATCACTACTTTAATGATCTAAACGTTTTTATATCAAATTATAGAAGAAGTAGTTGATAACCCGGCCGCCGCAGCGCGTACATCCTTTACAGCCGACATGAATATCATATCATGAATTAACTGACACAAGATACATTGGATCGCAAAGAACCGGAAGACCACAAATTAAGATCCATGATGAAACATCGATCACACCGTGTAATAAGCTGCCGACGGCAACAACCTGGGCACGGGCTGAAGCATTAGCGGCACGGCCCGCTGCAGGGTGAGACCATAGGCCTCCTCCATGTCGAGCTTTTGGGGCGTCATGCCGTCAACCAACGACCAGTCGAAGGCGTGCACGAGCGTGGCGGTCATGAGAGTGACCATTCGGAGACCCCAGCTGAGGCCCGCGCAGATCCTTCGGCCCGCCCCGAACGGAATGAGCTCATAGTCGGCCCCCTTGACGTCCACGCTCTCATGCGACCCACCCGAGAGGAAGCGGGCGGGCCTAAACTCTAGTGCGTCGGGCCCCCATGAGTCCGGGTCACGGGCGATGGCCCACACGTTGACGAGGAGGGTGGTGCCCTTGGGGACCCGGTAGCCATCCACCTCACAGTCCTCTGCGGCCACCCGGGGAAGGGAGAGCGGCGTCGACGGGTGCAATCGGAATGTCTCCTTGATGACGGCGGCGAGGAAGGTGAGCTGAGGCAGGTCGGATTCTGTGACGAGGCGCTCATTTCCGACAACGTCGTCCAGCTCGCGCTGGAGCTTCTTGAGGACGTCCGGGTGTCGTATCAGCTCCGCCAGCGCCCACTCCACCGTGCTCGACGTCGTGTCCGTCCCCGCGGTGAACAGATTCTAAAACAAGGAACATGTTTCAAGTCTTTTTTTCTTTGGGAAGAACGACAAAACCACATTTTTTAGTTAGGCGAAGCAATAAACACATTTTTGAGTAGTACGTACCAGGAGTAGAGCTTTGATGTCAGTGTCATTGAAGTTGATCCCTTGGTCGTCGTCGGGCGGCGAGTGGCGCATCATCCCGAGCATGACGCTGAGCAGGTCATTCCCCTGGGCGTCGGCCAGGTGCTCCCTCTCGCTGATGAATCCGTCCATCATGCGGTCGTATCGGCGGTGGAGGCGCTTCATCTTGCCGACGACGCCCTGCGGGTCGAGCCACCGGAGCGCGGGCACGAAGTCGCCGATGTTGAAGACGCCTGCGAGCTGCATGAGCTCGACGACCATGTCCTTGAACTCCCTGGCGCCCTCGCCGACGCCGTGCCCGAAGACGCGGCGTCCGACGGCGGCGAGCGCGAGCGCGTTGGTGGCGCAGACGTTGGCCTCCTGGCCGACGGCGACGGGCAGGCCCGAGGAGGAGGAGAGGAGGTGCAGGCGGGTGACCATGAGGCGGGCCTCTTCCTGGCGGACGTAGCGCAGGGCGTCGAGCGCGCGGGCGGAGAAGAGGTGGAGCGCGCAGAGCTTGCGGAGGGCGCGCCAGCGGGCGCCGTAGGGGGCGAAGACGAGGTCCTGGTAGTTGTATGCGACGTGCTCGGCGCCCGAGTTGGGCGGGCGGTCGGTGAAGTTGGCGTCGTGGGCGCGGAGGAAGGTGGCGGCGACCTTGGCGGAGGCGGCCACGACGACGTCGGCGCTGCCGAAGCGGAGGCGGAAGAGCGGGCCGTGGCGGCGCGCGAGGGCGGCCATGGTGTGGTGCGGCTTGTCCCCGAGCTGCGGCAGGTTGCCCAGCACCGGCCAGCCCCTCGGCCCCGGCGGCAGCGGCAGCACGTTGGGTCCGTTGGCATGGCCGCGGAGGTGCCAGACGGCGGCGGCGAGGACGGCGGCGGCGAGGGAGGCGAGGAGCAGGAGCAGGTCGTGGTCCATGATGGCTTGTTGGTTAGCTGCAGGCGCGGGCATGCAGATGCAGATGCAGATCATGAGAGTGCACTGCAGTGTTTGTTAGGTTAGGCTCCATTCCATGCATGCATATATATAAGGAAATGGGAGGTCGTCGCCGCGTGCTCCACCTAAATCGCCGGATCCACCGGTCCTCCTACCTAACCCCTGCCCCTGCCGGAGGAGGAGGAAGAATGGCACTTGGCACCTCACCTCACCTCAGTAGTTGTATTTGTATTTGTACGAGTGGCAAGCAAGGAAACAAGACAAAAAACTGAGCTTTCTTTCCAATGGCTTTTGAGCTAGCTATATATTGACAAAGAAAGAAAGAAAGAAAGAGAGAAAGGAAGAACCTGCACTTGCCACGGTCCACCGTCCTAGGAAACAAGACAAACGACTCACCTTTCCAATCACTCACTCAGCAGCCACATATGTTAACTACTCACGGTTCACGGCACAAAAAACTACCACGTTTGAGTGAGTGAGCGATGAGTAAGCTACTCACGGTGGCACAAACTCGCACGTTCAGTGTAGCTTTCTTCCGCAAGCGGCACTTACTTTTTTTTTTAAACAATCACGGGAAGGAAAAGGTTCCTCACCCGAATATATTACTCAAAGGTCCGAGGACAGGTTACATCATAGATTAAAACGAGAGGAGAGGTATAGGCGCCAATATATGATAGCCTTCCTACTAAGTAGGTCCTTCAATCTTAAGACCCAAAACGTAAAGTCCGAAACAAGGTTCCGATCGAAGAGTAACTAGGGGTGTGCGTAGCTCGTTTTGAAAGACACGAACATTCCTGAAGTCCCACGGCTTCCAGAGGATGACGAGGGCAACAGTACGCCAAATGTTGATGTCGAGGCCGACTGGTGTCGTAGTCTCCCAAAGGCGGTCGATGGAGTGAGGAGGGCCCATACCTGAACCGCGTATGGGCAAAGGATGGAGATGTGTAATGCGTCCTCATGAGTGAGAGCGCACCGCGGACAATCCGAATAAGAGGTAATGGTCTTATGATGTAAGTTCGCCATCGTGCTCAACCTGTCACGGCAGTGAAGCCAACCGTAGATTCTGACCTTTATAGGTGCCTTTGAGCTCCAGATGTACCCATCATTGAGATCCATCTCGTGGTCGGAGGAGAGAAACGAGTAGGCGCACCTTGAGGAGAAGAAGCCGTGGGTGAGGAACCTGTCATCGGGCGCGTCGTTCGTGGCAACATTGCAACAAAGATAAAATAGACACAAGCTCTACAGAAGCAACAATGGCAAGGCGGTACCGCATAGTAGCGAGTAAACCGTTATGCATGACTTGTGATACCAGGACAGAGTGTCACGCTCAAGATGTGACCCTATCCTGAATTTGGCACGAAGGCCTCATCAGGGNNNNNNNNNNNNNNNNNNNNNNNNNNNNNNNNNNNNNNNNNNNNNNNNNNNNTACTGAAAAGAAGAGATATATATGCATCACTTGTGATACAAGTGTTGGGGAACGTCGCATGG

**>barke_contig_2826620 CAJV012632771 carma=1H**

CTCTTCTCCTCCCGCGCGCTCGACGCGCTCCGCTACGTCCGCCAGGAAGAGGCCCGCCTCATGGTCACCCGCCTGCACCTCCTCTCCTCCTCGGGCCTGCCCGTCGCCGTCGGCCAGGAGGCCAACGTCTGCGCCACCAACGCGCTCGCGCTCGCCGCCGTCGGACGCCGCGTCTTCGGGCACGGCGTCGGCGAGGGCGCCAGGGAGTTCAAGGACATGGTCGTCGAGCTCATGCAGCTCGCAGGCGTCTTCAACATCGGCGACTTCGTGCCCGCGCTCCGGTGGCTCGACCCGCAGGGCGTCGTCGGCAAGATGAAGCGCCTCCACCGCCGATACGACCGCATGATGGACGGATTCATCAGCGAGAGGGAGCACGTGGCCGACGCCCAAGGGAATGACCTGCTCAGCGTCATGCTCGGGATGATGCGCCACTCGCCGCCCGACGACGACCAAGGGATCAACTTCAATGACACTGACATCAAAGCTCTACTCCTGGTACGTACTACTCAAAAATGTGTTTATTGCTTCGCCTAACTAAAAAATGTGGTTTTGTCGTTCTTCCCAAAGAAAAAAAGACTTGAAACATGTTCCTTGTTTTAGAATCTGTTCACCGCGGGGACGGACACGACGTCGAGCACGGTGGAGTGGGCGCTGGCGGAGCTGATACGACACCCGGACGTCCTCAAGAAGCTCCAGCAGGAGCTCGACGACGTTGTCGGAAATGAGCGCCTCGTCACAGAATCCGACCTGCCTCAGCTCACCTTCCTCGCCGCCGTCATCAAGGAGACATTCCGATTGCACCCGTCGACGCCGCTCTCCCTTCCCCGGGTGGCCGCAGAGGAGTGTGAGGTGGATGGCTACCGGGTCCCCAAGGGC

**>bowman_contig_404 CAJX010000403 carma=1H**

TAGTGCGTCGGGCCCCCATGAGTCCGGGTCGCGGGCGATGGCCCACACGTTGACGAGGAGGGTGGTGCCCTTGGGGACCCGGTAGCCATCCACCTCACACTCCTCTGCGGCCACCCGGGGAAGGGAGAGCGGCGTCGACGGGTGCAATCGGAATGTCTCCTTGATGACGGCGGCGAGGAAGGTGAGCTGAGGCAGGTCGGATTCTGTGACGAGGCGCTCATTTCCGACAACGTCGTCGAGCTCCTGCTGGAGCTTCTTGAGGACGTCCGGGTGTCGTATCAGCTCCGCCAGCGCCCACTCCACCGTGCTCGACGTCGTGTCCGTCCCCGCGGTGAACAGATTCTAAAACAAGGAACATGTTTCAAGTCTTTTTTTCTTTGGGAAGAACGACAAAACCACATTTTTTAGTTAGGCGAAGCAATAAACACATTTTTGAGTAGTACGTACCAGGAGTAGAGCTTTGATGTCAGTGTCATTGAAGTTGATCCCTTGGTCGTCGTCGGGCGGCGAGTGGCGCATCATCCCGAGCATGACGCTGAGCAGGTCATTCCCTTGGGCGTCGGCCACGTGCTCCCTCTCGCTGATGAATCCGTCCATCATGCGGTCGTATCGGCGGTGGAGGCGCTTCATCTTGCCGACGACGCCCTGCGGGTCGAGCCACCGGAGCGCGGGCACGAAGTCGCCGATGTTGAAGACGCCTGCGAGCTGCATGAGCTCGACGACCATGTCCTTGAACTCCCTGGCGCCCTCGCCGACGCCGTGCCCGAAGACGCGGCGTCCGACGGCGGCGAGCGCGAGCGCGTTGGTGGCGCAGACGTTGGCCTCCTGGCCGACGGCGACGGGCAGGCCCGAGGAGGAGAGGAGGTGCAGGCGGGTGACCATGAGGCGGGCCTCTTCCTGGCGGACGTAGCGGAGCGCGTCGCGCGCGCGGGAGGAGAAGAGGTGGAGCGCGCAGA
