# Supplementary material for: Regulation of the Flavonoid Biosynthesis Pathway Genes in Purple and Black Grains of Hordeum vulgare
Source: PLoS One. 2016 Oct 5;11(10):e0163782. doi: 10.1371/journal.pone.0163782 (PMC5051897; doi:10.1371/journal.pone.0163782)
Supplement: S2 File — Reconstructed full-length nucleotide sequence of Ans is also present. Yellow color marks coding sequence of the gene. (DOCX) [file pone.0163782.s013.docx]

**S2 File. The nucleotide sequences of Bowman contigs corresponding to the *Ans* gene found in BARLEX database. Reconstructed full-length nucleotide sequence of *Ans* is also present.** Yellow color marks coding sequence of the gene.

**>bowman_contig_1993730 CAJX011988163 carma=5HL**

CCGACGACGGCCAGGCTGGCCGGGGAGGCCGGGTGCGCGGCGTCGAAGGCGGCCACGTCAACAACGGGGATCTGCGGGGAGCCCGCGGCGGACCAGCTGGCGGCCGCCTCATCGTAGGCGTCGCCCAGGCCCTGGCGCTCCTCCTGCGGCCGCACGTACTCGGCTGGGATCGCCGAGAGGCCGCTCATGCTCAGCGCCTCCACCCGCGCCATCTTTTCTTCTTCCTCCTCCGGTGGTATGCTCTGATTTGAGTCACTTTGCTTGGTTGCTGATCGGGGATCGGAATGGATGGAGTTCATGGATGGAATGGGTTTAGGGTTACTTATAGATTGTTTGGTGGTTAGCTCCAACTTTCCATCACGCGCGCGCGCGCGCAGAAGCAGAACAGCAGAGTTCGCTTCCAACCACACGCACGTGCTGCATGCTACCGTCCAAGTTCCAACTCCATTCGTCTTCTTCTTATCTGACCACAAAAAGGAAAAGGAAAAAAAAAACAGACAAACAAACAGTTTGGGCCAGGGATGGTGGGCTGAGATGCGGACACGAGAGGATGAGTTGCAGGTCCAGAACAAAAGTTGAAAACATTTTGACACTTCCTAAAAAAATATAAAAGCACGAATATCTGTCAAATTTGTGAACACATGTTCTTTATAAATGATTTTTTTTAAATGCGAACATTGTTTAAAAACAAATTTGTGAACAATTTTTTTGAATTACCGAACATTTTCTGAAAACAAGTGAACAAATTGGAAATCCGGTTTTCATGGAATTTACGAACAAAATTTGAAAACGGGAGCATTTTCTTTAGAAAGGATCTTTTTTTTAAATGTGAACATTTGTTTAAAAATAAAAATAAATTTAAGAAATAAATAACCAGAAAAAGAAAAAAAGAAATAGAAACAAAATAAAAGAGAAACAAAACACACAACCATTCCGGCTCAGGAATCTTTTAGAAGTTTTCCAAAACCGTAAAAGATGGCTGGGAAGCTCTAAAAGGTTCCCGAAACCGAAAAAGCTGAAAACATTTAGATGGGTCGACACAATCTAAACGATCGATGGGTCACATGTACAATCAGTCCATGGCCCATAGTTTGACGCAGAGAGCGTTAAATAGAATTTACCCCTAGCATGGCCCAGCACATTGCACTGTTGACATGCCCACGACCGGTTGACTAGAAACAGGAATTTTCTAATCGAATATCTTCGGTCTTGCTTGTTTTTAGAAAAGGAGATTAAAAACCTGGCCTCTGCATTAATCGATGCATATATTCATCTTTATTCATAATTCAACATAGAGATAACAAAAACATACATCAAACCATCTTAAGCCATCACTCACACCTACAAAACTCGATAATGTTGGGGAATGTCACATGGAAAACAAAAAAAATCTTATGCTCACCAAGATCAATATATAGAGATCAACAACCACGAGAGGGAGAGTGCATCTACATACCATGTATATCTCTAAGCGAAAGCGTTAAGAAACGCGGTTGATGTAGTCGAACTCTTCGCGATCAAATCATGATCCGTGCCGCGGTCCGATCATGAACGTCACAATCAAGTGCCGGACAGACGGCACCTCCGCGTCCAACACACGTATAGCTCGATGATACCTTCACCTCCTCGATCCAGTGAGCGATGGTGAATTAGAGGTTCGTGGCTGCAATGCTGGTGGAGCAATCTCGGCAAGGCTTCACCAAGCGCTGCCGCGATCACGACGGTGGAGAAGTACTATGAGAGAGAGAGAGGGAAGGGTCGTGGCTGTTTTCAGGTGCGGCTGCCCTCCCTTTCCTCTAGTATATATAGGAGGGAGGGAGAAAGGGTGGCGCCCTAGGGTTTCCCCTAGGGTGGGTTGCGGCCACCTAGGTGGCTTGGCCACCAAGCTAGGTGGGGCGACGACCATCTCAGGGCCAGCCCACCACCCTGGCCGCATGGGCCTTAGGTGGGAGGGGCTGGTGCGCCACTTCCCACAGCCCATGGGGCCCTCCGGGATAGGTGGACCCTCCCAGTGGACCCCCAGAACCCTTTCGGAACCTTCGTTACAAAATCGGTAACCTTCCAGAACTATTTCAGAACTCGGATACCAACTTCACATATATGAATCTTTACCTCCGGACCATTCCGAAGCTCCTCGTCACGTCCGGGATCTCAT

**>bowman_contig_941389 CAJX010936364 carma=5HL**

GGGATCCGCCGGCGAGGACGGGAAGAGGGAGTGGGAGGACTATTTGTTCCACATGCTCCACCCCGACGCCCGCGCCGACCACGCGCTCTGGCCGGCGCATCCGCCGGAGTACGTGCCCGTCACCAAGACCTTCGGGGAGCGCGTGAGCGAGCTCGCGTCCCGGCTCCTCGCCATCCTCTCCCTCGGGCTCGGCGTCCCGGCCGACACCCTCGAGCGCCGGCTCCGCCTCACCTCCGGCGACGCCGAGGTGGAGGTGGAGGACGACCTGCTGCTGAAGCTCAAGATCAACTACTACCCGCGGTGCCCGCAGCCGGAGCTGGCCGTGGGCGTGGAGGCGCACACCGACGTCTCAGCGCTCTCCTTCATCCTCACCAACGGGGTGCCGGGCCTGCAGGTCGTCAACAGCGACGGGGCATGGGTCACCGCGCGCGACGAGCCGGGCACGCTCGTCGTCCACGTCGGCGATGCGCTCGAGATCCTCAGCAACGGGCGCTACACCAGCGTGCTCCACCGGGGGCTCGTCAACCGGCAGGCTGTGCGCGTCTCCTGGGTCGTCTTCGCCGAGCCGCCGCCCGACTCCGTGCTGCTGCGCCCGCTGCCGGAGCTCCTTGTGGACGAGCCGCCACGCTTCGAGCCGCGCACTTTCAGGCAGCACCTCGAGCGCAAGGTCCTCAAGAAGAATGATGCACAGGAGGAGGAGGAGGTGAAGAAGCCGCCCGTCGCTGCTCAAGAGGAGGAGGAGGAGGCCTCGAAGCCGCTGGTCGCCGGCGAGGAGGAGCACAAGGTCCTGAAGGAGTAGAGCGAACAGGTAGAGGCCATGAAGCCGCCGGTCGCCGGCGGGGAGGAACACAAGGTCGTGAAGAAGGTGCAGAGTGAACAGCAGGAGGCCAAGATTGGAGAGGCGCCGGTGGAAGTTAATTAAATATATATATACGAATATAGGATACTGGGAGAAAGGAGTGCATTATAAGCAGGAGTATTATGTATTCACATTGAATTAATGGGAAATCTAAATTATGTACCATGCACCGCCGTCCTCTCGTGCAGCGGCTCGGACGCCACACGATTCCTTTCAGTTGCAGTAGTTTTCCTACAATACGTAATGAGTTGTTTTTCTCACATAGGTCTGTAGCTAGCAGTCGAATGTTACAATGCATATTTCTAAGATCATAACTGTTTTTTCTACAGCATACAAGCATGTAATGATGTCATCAAGATTTCCTTTGATGTTTTGTAATTTTAAAAAAAATCTATAAATCGTTAATTCAATCGATGATCCGTTTTTACCCTTGACTTTATCGCGACGAGTTCTTTGAAACTAGATCCCATGTCGACATGTTCCGTCAACTATTTTTTTCGTTCATACTTGTCATACACTAATAGAAATAAGGGCTTTCGTCCCAGCTCGAAAAACACATTAGNNNNNGTTCCTTTACGAACCGGGACTAATGTTAGTATTAGTCCCGGTTCG

**>bowman_contig_1630795 CAJX011625634**

CCAGCCTGGCCGTCGTCGGCGCCGTGCGCGCCGCCGCGGAGGAATGGGGCGTCATGCACGTGGCCGGCCACGGCATCCCGGAGGACCTCATCGACGCGCTGCGTGGCGCCGGCACGGGGTTCTTCCGCTTGCCGATCGAGGACAAGGAGGCCTACGCCAACGACCCGGCAGCGGGGAGGCTGGAGGGCTACGGCAGCCGGCTCGCGGGATCCGCCGGCGAGGACGGGAAGAGGG

**>bowman_contigs_941389_1630795_1993730**

atgagatcccggacgtgacgaggagcttcggaatggtccggaggtaaagattcatatatgtgaagttggtatccgagttctgaaatagttctggaaggttaccgattttgtaacgaaggttccgaaagggttctgggggtccactgggagggtccacctatcccggagggccccatgggctgtgggaagtggcgcaccagcccctcccacctaaggcccatgcggccagggtggtgggctggccctgagatggtcgtcgccccacctagcttggtggccaagccacctaggtggccgcaacccaccctaggggaaaccctagggcgccaccctttctccctccctcctatatatactagaggaaagggagggcagccgcacctgaaaacagccacgacccttccctctctctctctcatagtacttctccaccgtcgtgatcgcggcagcgcttggtgaagccttgccgagattgctccaccagcattgcagccacgaacctctaattcaccatcgctcactggatcgaggaggtgaaggtatcatcgagctatacgtgtgttggacgcggaggtgccgtctgtccggcacttgattgtgacgttcatgatcggaccgcggcacggatcatgatttgatcgcgaagagttcgactacatcaaccgcgtttcttaacgctttcgcttagagatatacatggtatgtagatgcactctccctctcgtggttgttgatctctatatattgatcttggtgagcataagattttttttgttttccatgtgacattccccaacattatcgagttttgtaggtgtgagtgatggcttaagatggtttgatgtatgtttttgttatctctatgttgaattatgaataaagatgaatatatgcatcgattaatgcagaggccaggtttttaatctccttttctaaaaacaagcaagaccgaagatattcgattagaaaattcctgtttctagtcaaccggtcgtgggcatgtcaacagtgcaatgtgctgggccatgctaggggtaaattctatttaacgctctctgcgtcaaactatgggccatggactgattgtacatgtgacccatcgatcgtttagattgtgtcgacccatctaaatgttttcagctttttcggtttcgggaaccttttagagcttcccagccatcttttacggttttggaaaacttctaaaagattcctgagccggaatggttgtgtgttttgtttctcttttattttgtttctatttctttttttctttttctggttatttatttcttaaatttatttttatttttaaacaaatgttcacatttaaaaaaaagatcctttctaaagaaaatgctcccgttttcaaattttgttcgtaaattccatgaaaaccggatttccaatttgttcacttgttttcagaaaatgttcggtaattcaaaaaaattgttcacaaatttgtttttaaacaatgttcgcatttaaaaaaaatcatttataaagaacatgtgttcacaaatttgacagatattcgtgcttttatatttttttaggaagtgtcaaaatgttttcaacttttgttctggacctgcaactcatcctctcgtgtccgcatctcagcccaccatccctggcccaaactgtttgtttgtctgttttttttttccttttcctttttgtggtcagataagaagaagacgaatggagttggaacttggacggtagcatgcagcacgtgcgtgtggttggaagcgaactctgctgttctgcttctgcgcgcgcgcgcgcgtgatggaaagttggagctaaccaccaaacaatctataagtaaccctaaacccattccatccatgaactccatccattccgatccccgatcagcaaccaagcaaagtgactcaaatcagagcataccaccggaggaggaagaagaaaagatggcgcgggtggaggcgctgagcatgagcggcctctcggcgatcccagccgagtacgtgcggccgcaggaggagcgccagggcctgggcgacgcctacgatgaggcggccgccagctggtccgccgcgggctccccgcagatccccgttgttgacgtggccgccttcgacgccgcgcacccggcctccccggCCAGCCTGGCCGTCGTCGGCGCCGTGCGCGCCGCCGCGGAGGAATGGGGCGTCATGCACGTGGCCGGCCACGGCATCCCGGAGGACCTCATCGACGCGCTGCGTGGCGCCGGCACGGGGTTCTTCCGCTTGCCGATCGAGGACAAGGAGGCCTACGCCAACGACCCGGCAGCGGGGAGGCTGGAGGGCTACGGCAGCCGGCTCGCGGGATCCGCCGGCGAGGACGGGAAGAGGGAGTGGGAGGACTATTTGTTCCACATGCTCCACCCCGACGCCCGCGCCGACCACGCGCTCTGGCCGGCGCATCCGCCGGAGTACGTGCCCGTCACCAAGACCTTCGGGGAGCGCGTGAGCGAGCTCGCGTCCCGGCTCCTCGCCATCCTCTCCCTCGGGCTCGGCGTCCCGGCCGACACCCTCGAGCGCCGGCTCCGCCTCACCTCCGGCGACGCCGAGGTGGAGGTGGAGGACGACCTGCTGCTGAAGCTCAAGATCAACTACTACCCGCGGTGCCCGCAGCCGGAGCTGGCCGTGGGCGTGGAGGCGCACACCGACGTCTCAGCGCTCTCCTTCATCCTCACCAACGGGGTGCCGGGCCTGCAGGTCGTCAACAGCGACGGGGCATGGGTCACCGCGCGCGACGAGCCGGGCACGCTCGTCGTCCACGTCGGCGATGCGCTCGAGATCCTCAGCAACGGGCGCTACACCAGCGTGCTCCACCGGGGGCTCGTCAACCGGCAGGCTGTGCGCGTCTCCTGGGTCGTCTTCGCCGAGCCGCCGCCCGACTCCGTGCTGCTGCGCCCGCTGCCGGAGCTCCTTGTGGACGAGCCGCCACGCTTCGAGCCGCGCACTTTCAGGCAGCACCTCGAGCGCAAGGTCCTCAAGAAGAATGATGCACAGGAGGAGGAGGAGGTGAAGAAGCCGCCCGTCGCTGCTCAAGAGGAGGAGGAGGAGGCCTCGAAGCCGCTGGTCGCCGGCGAGGAGGAGCACAAGGTCCTGAAGGAGTAGAGCGAACAGGTAGAGGCCATGAAGCCGCCGGTCGCCGGCGGGGAGGAACACAAGGTCGTGAAGAAGGTGCAGAGTGAACAGCAGGAGGCCAAGATTGGAGAGGCGCCGGTGGAAGTTAATTAAATATATATATACGAATATAGGATACTGGGAGAAAGGAGTGCATTATAAGCAGGAGTATTATGTATTCACATTGAATTAATGGGAAATCTAAATTATGTACCATGCACCGCCGTCCTCTCGTGCAGCGGCTCGGACGCCACACGATTCCTTTCAGTTGCAGTAGTTTTCCTACAATACGTAATGAGTTGTTTTTCTCACATAGGTCTGTAGCTAGCAGTCGAATGTTACAATGCATATTTCTAAGATCATAACTGTTTTTTCTACAGCATACAAGCATGTAATGATGTCATCAAGATTTCCTTTGATGTTTTGTAATTTTAAAAAAAATCTATAAATCGTTAATTCAATCGATGATCCGTTTTTACCCTTGACTTTATCGCGACGAGTTCTTTGAAACTAGATCCCATGTCGACATGTTCCGTCAACTATTTTTTTCGTTCATACTTGTCATACACTAATAGAAATAAGGGCTTTCGTCCCAGCTCGAAAAACACATTAGNNNNNGTTCCTTTACGAACCGGGACTAATGTTAGTATTAGTCCCGGTTCG
