# Supplementary material for: Regulation of the Flavonoid Biosynthesis Pathway Genes in Purple and Black Grains of Hordeum vulgare
Source: PLoS One. 2016 Oct 5;11(10):e0163782. doi: 10.1371/journal.pone.0163782 (PMC5051897; doi:10.1371/journal.pone.0163782)
Supplement: S3 File — Exonic sequences are marked by green color. (DOCX) [file pone.0163782.s014.docx]

**S3 File. The nucleotide sequence of Bowman contig corresponding to the *Ant2* gene found in BARLEX database.** Exonic sequences are marked by green color.

atgtcgcatgagagccattacaaccaacgacgtcgcatgagagataacacaactcaacgacgtcgcatgggagctattcgcccgtcaaaaatgcaatgcatgtactccgatttagacgaaaaattatccaacttttaaggaagcagttcctctggtttgtttaaatttgcatcaattttgtttattctgttaaattccgttcgaaatgtcgtcatatatttacgactatggttggatggtcagctactatctgcgaattgatcctcacctgtcggcgcacagatgtggtgtcggattttggtgtgggcgatggagatgcccttaaggcggaaacctgtcgacctacgtgttccaatccccactaacctccttttgattttcactgagccaaccacctcatctgtaaatccagttcgtaatttctggattccttatgatgccgatggtagattccttggcaaaggaggtgctggaacacacgtacaagactttagcagttactccctctgtaaactaatataaaaacatttagaccactactttagttatctaaacgctcttccattagtttacagatggtgtaactatagaccgcatcgacagtgatgctcgccgtgtgtttccttagttgtcgttgacgttgattattctgtgcctgttaccccatcacctcagcgtcaagcagagatcggtgaggattgcgtacatctcactccatttttatcatagttttaaatagccggctatagtcccgctataaccttttcaatagggtgccgctaaatggtatcacgtacaaatatgccgctatagcccgatatagcccgctatagctccgctatagctgattttcaggcatgccgctatttgtcatagcccgctatttaaaacattgattttaactaagcactgagaagaccaagcaggcatgcacaccaaactaatcagaaagtgatgggcctaatatgaaactaatcagaagtaccatgttggagcactacagtagtgtccggacagtactttgcttttgcgtcgcagtctcgcttgttgttggctcgttgctcggagcctcggacagatctatataacatggcttggattctagatacattgcacatatagaagctccttcttcctctccggacgacaggttggactccgggcttcttggtctccatagctcaaagtaaacctttaacctcctcttagatggaaaattaatcctccactcatgtctagcttagtttatctcttactttcttggtttgttccagctttcgatctagtatatacagtatgttctttgtaatcttcgttgagaaatcttcccaggagtttcttaattagcaccagtgcaacagagtggagggatttcgatcagctagcatgaactaaattatccaagaagatgcatcatgctcactataaacacaagagatggaaccatatatagcttcattgatttgtttatttttgcctccttgcttgctctaacttcacctcgtctgcatcatgcatatgaaatgaaggaaggaaataatatggtaatggcgctaccaatagttcgtccgagccaggaagaaccgccgacggggaagcaattcagctaccagctcgctgccgctgtgaggagcatcaactggagctacgccatattctggtccatttcaaccagccgtccagggtagggagtgcatcagattgatgatcacttggcactggccgtttcctttcctcttatgatccgtttgttatgtagggtactgacctggaaggacgggttctacaacggcgagataaagacgaggaaggtcaccagctcggcggacctcaccgccgaccagctcgtcctgcagaggagcgagcagctgcgggagctctaccagtccctcttgtccggccagtgcgaccaccgggggaggcgcccggccgccgcgctctcgccggaggacctcggggacgccgaatggtactacgccgtctgcatgagctatgccttccgccctggccaagggtatgtactactccgtagtatgcttcaagaacaactcctctgatccacaagataacatttatcaatagttaacaatcatcttatcttgataagatgagagttattttgctagagagtgaaaactacaacgtttgctatttcgtcatcaggttgccaggcagaagctttgcgagcaacgagcctgtttggctgtgcaatgctcagtgcgcagacaccaaaactttccaacgctcgctcttagcgaaggtacgtttgattgcaccgcccgagtgtctcgcaagcctcactctcatgtagtccctccgtaaataaatggagtataagagtgtttagatcactacttagtgatctaaacgcttttatgtttctttacgaagggagtactatctgcctaatatgtacccgatcttgactaataccatgttgtctactgtgtacttttggcgcacatactatatctgtcggctaaatcagacgacgtctatccaggttagctagcgcatgtcatgcaccctatatctgtgtatactgttcatgtttctgaccccagctgcacgggtgacaattttttttcttcagacggtcgcctgcattcccttgatgggtggtgtgcttgagctcgggacgacagataccgtgagctttcctcgcatcaagcacatgatattcacttacggtttaaaattaaacggaaaactcactattagtaacgctttgtcgcatcaagcacatcgaaattgcaaaatttaacccgggttccatggagccttgttgttaagaaaaacacaacagaagttgtgagcacctatatatttccgttcagtacatcttgccatatgtgggtagacaacaaagacaaatatgaggatctacaaatagccaataatagatgtgctagtatttccaaaactacacattgtcatcttgtgtagctcacatgcatgtgttatagtgtttattatcgaatgctacgtatatgctagtatagatctatatatacatctaggtagacttgttttactgttttttttcaaagttgtaaatattatttgattttttttccaaaatttcagtctcactacaaccctgatctcccaattattcgctcaagctgccatgtcaactatggtacaagtttgaagtttgttaccctttcattctttccctgcccttgttgcttacaaatgcgagctttgctacaagtacggacgttttacaggatttttacaggcaaagttgaagtggcttattttaattgatacttagatgaggccggccctaaaaatcaaggggagattagtgaggagaaaaatccatcagtctgtaaccttctgtaaaaaaaaggtccgtaggtctggcattttcctacaaatgcggacgaaaccaatctcttttgaggcctctgctacgttattctagtgtgttatttttcaagaacaagaaacacaatgctgcatgtcattgtcattaagaaggaaagggagttaattagaagcaaatagtcgtgacaaagcatgtagtgttgttataccacacgtgagaccaccgccaccacattcatatctaggcccactcctccctaatctttgtcactgactgcctagcctttttggaaagcataagttcttgtgccgccggtgcgaccactctttgcaccgatgacactttcccatcaaaaactctagtgttgcgctctagccttcattaatgtcagccagccggtcttgaacgacaatgaagtcttccaagtcgcccacaacgtgaggttgcaaatctactttggtgaaggcacatgtgcgtatagccccaaatacgtgcacagaaatttggccacttgatttgcaagagaagcggtcagaacatccagacaatccactccctcgcatcgggtaggtgaaatcacatgtttctaaagattaaccacaagaccaaaatcgccaccaaataacgtcaaagatctccttccttgccaacaactcgctttctctgggtttgaggtaacccgatagccggtgacgaacaccccatctatccaaaggttcagacacaccaaaatcctccactttattccttgtgcaggtggtaaagccccttgcctcgtggcatgacaataggttgtcctgattttgtttctcggattcgtcataaaggggaggcttttataccgtatgattgaaaattttgtattctatcagtaaattacgataaataaataaaaatacttcttttaatgtaaaatatattcttatactatgtgacaatgggaaggctgtaccgtttgctcgggacactcattaagtcatgaagacacaatactactaaatcaatccatgaattgtttgagttcttcaccaaattaaatgctactgtgctgattaattcattcgaatataagttaaaaattaaccaaagcactatcaatcgatttgctaggttttggaggacagagacatggtgaaccgaatcagcacatctttctgggacctgaagatcccaacaagctcgaagccgaaggagccctccagcccatcagcagacgacgctggtgaggccgacatcgtgttccaagacctcgaccacaacaccatggccgcgatgatccccggggaactcgagctaggggaggtcgagtgcctgtccgacgacaacctcgagcggatcacgaaggagatcaacgggttctacggcctgtgcgacgagctggacgtcggcgctctcgacgaaaactggatcataggcgggtctttcgaggtcatgtcctcgccggaagcgccaccggcgcctgcagccaccggcggcatcactgatggtattgtcactttaagcgccgctgcgtcctctctctcatcgtgctttacggcttggaagagatcgtgggactcagccgaagacatggctgcgccggtcgccgggcagtcgcagaagttactgaagaaagctttggccggtggtgcgtgggcgattaacggcggcggcggcggcggcacggcgagagctcaggaaagtagcaacaccaagaaccatgtcatttcggagaggcggcgccgggagaagctcaacgagatgttcctgattctcaagtcactggtgccgtccattcacaaggtaacacgcgcacatcaccggaaagcaaaagaaaaatactgtccagagatgtgtctctgaacttttgtttgcaggtggacaaggcatccatcctagctgagacgatagcctatctcagagagctggagcaaagggtggaggagctagaatccaacagggcgccgtcgcggccggccggagcagccgtccggagacaccatgacgccgcggcgaagaagatgttagctggatccaagagaaaggcgtcggagctcggcggggacgacggcccgaacagcgtcgtcaacgtcacggtgatggagaaagaggtgctcctggaggtgcaatgccggtggaaggagctgctgatgacgcaggtgttcgacgccatcaagagcctccgcctggacgtgctctccgtgcgcgcgtcgacgcccgacggcctcctcgctctcaagatacgagctcaggtccgtctggtagcagctgattatttaagctccatccggcggtaaaaatgcagcattaattaacgctcgtatgcatggctatggtttactgttgcagttcgccggtcctggcgccgtggagcccgggatgatcatcggagcgcttcagacagctacacgagggcgctgaaaaagaagcatgctgctgagacactggtactgtactgtagatccgcgtcacgagctgggacatacatgaaagattgacaatgatggtggctatcttcttcggacatgacaattattttcattcgttccgatgtgcagtgatgtgggacgtgacaaatagatttccgatctttggatccacttgaaatcatgtgaaccgcagagctcgtgggtgcaggatgcgcgtcacgagcgcgcgcggtgcaactaccagtctccccacatgccggtcgtacaacggtgtatggcgcgggttgctcctcacctgacatcgcatctgtagactgtagcaaatctggacggcgcgacgggataccaccgcgagcgagcgcccggtcggccccgctcgctcggctgcgcgacagcgcgcgtgcgccggcgacgccgactggccagctcgtgctcgcccatgtcgcggccatacacgacgcaccgctgcgcgtcatgtaaccttttcctccgtactacgttcccgtcggatgcgcatgcgccgtgacgacatggccgaaggaattttaacgtggtccttcttgcctcatctctactattaaaagcacgaaaggcggatccaactcactatccaaaacgtctaatgagatgatcaatggattaaaatagcctgttaacgaaagtgatttaccaac
